# Stem Cells Inhibition by Bevacizumab in Combination with Neoadjuvant Chemotherapy for Breast Cancer

**DOI:** 10.3390/jcm8050612

**Published:** 2019-05-06

**Authors:** Renaud Sabatier, Emmanuelle Charafe-Jauffret, Jean-Yves Pierga, Hervé Curé, Eric Lambaudie, Dominique Genre, Gilles Houvenaeghel, Patrice Viens, Christophe Ginestier, François Bertucci, Patrick Sfumato, Jean-Marc Extra, Anthony Gonçalves

**Affiliations:** 1 Department of Medical Oncology, Institut Paoli-Calmettes, 13009 Marseille, France; viensp@ipc.unicancer.fr (P.V.); bertuccif@ipc.unicancer.fr (F.B.); extrajm@ipc.unicancer.fr (J.-M.E.); goncalvesa@ipc.unicancer.fr (A.G.); 2 Aix Marseille Univ, CNRS U7258, INSERM U1068, Institut Paoli-Calmettes, CRCM, 13009 Marseille, France; jauffrete@ipc.unicancer.fr (E.C.-J.); lambaudiee@ipc.unicancer.fr (E.L.); houvenaeghelg@ipc.unicancer.fr (G.H.); CHristophe.ginestier@inserm.fr (C.G.); 3 Department of Biopathology, Institut Paoli-Calmettes, 13009 Marseille, France; 4 Department of Medical Oncology, Institut Curie, Paris & St Cloud, Université Paris Descartes, 75005 Paris, France; jean-yves.pierga@curie.fr; 5 Department of Medical Oncology, Institut Jean Godinot, 51100 Reims, France; hcure@chu-grenoble.fr; 6 Department of Surgical Oncology, Institut Paoli-Calmettes, 13009 Marseille, France; 7 Department of Clinical Research and Innovation, Institut Paoli-Calmettes, 13009 Marseille, France; genred@ipc.unicancer.fr; 8 Department of Clinical Research and Innovation, Biostatistics unit, Institut Paoli-Calmettes, 13009 Marseille, France; sfumatop@ipc.unicancer.fr

**Keywords:** early breast cancer, bevacizumab, neoadjuvant chemotherapy, cancer stem cells, ALDH1

## Abstract

Preclinical works have suggested cytotoxic chemotherapies may increase the number of cancer stem cells (CSC) whereas angiogenesis inhibition may decrease CSC proliferation. We developed a proof of concept clinical trial to explore bevacizumab activity on breast CSC. Breast cancer patients requiring preoperative chemotherapy were included in this open-label, randomized, prospective, multicenter phase II trial. All received FEC-docetaxel combination, and patients randomized in the experimental arm received concomitant bevacizumab. The primary endpoint was to describe ALDH1 (Aldehyde dehydrogenase 1) positive tumor cells rate before treatment and after the fourth cycle. Secondary objectives included safety, pathological complete response (pCR) rate, disease-free survival (DFS), relapse-free survival (RFS), and overall survival (OS). Seventy-five patients were included. ALDH1+ cells rate increase was below the predefined 5% threshold in both arms for the 32 patients with two time points available. Grade 3 or 4 adverse events rates were similar in both arms. A non-significant increase in pCR was observed in the bevacizumab arm (42.6% vs. 18.2%, *p* = 0.06), but survival was not improved (OS: *p* = 0.89; DFS: *p* = 0.45; and RFS: *p* = 0.68). The increase of ALDH1+ tumor cells rate after bevacizumab-based chemotherapy was less than 5%. However, as similar results were observed with chemotherapy alone, bevacizumab impact on breast CSC cells cannot be confirmed.

## 1. Introduction

Breast cancer remains the first cancer in women in western countries [1]. Breast tumors can benefit from neoadjuvant chemotherapy to enhance the rate of conservative surgery [2,3,4,5]. Preoperative systemic treatments have also an impact on micro metastatic disease and have similar survival results than adjuvant systemic treatments [5,6]. It is also a way to assess the efficacy of new drugs or combinations and to develop translational research programs [7,8]. Major regimens used in this setting are sequential combinations of anthracyclines and taxanes [9,10,11,12], with the addition of trastuzumab and pertuzumab for HER2 (human epidermal growth factor receptor 2)-positive tumors [13,14].

Neoangiogenesis is a well-known hallmark of cancer [15], highly involved in tumorigenesis [16], and balanced by activating and inhibiting signals. Dysregulation of the equilibrium leads to an “angiogenic switch” allowing a tumor to grow over one to three mm [17]. The main pro-angiogenic and most specific factor is the vascular endothelial growth factor (VEGF). VEGF proteins are released by tumor cells, bind to their receptors (mainly VEGFR1 and VEGFR2), and are involved in cell division, endothelial cell migration, extracellular matrix modifications, vessels permeability, and vessels survival [18,19,20]. A combination of chemotherapy and an anti-VEGF monoclonal antibody (bevacizumab) has been shown to improve progression-free survival in first-line metastatic breast cancer [21,22,23] as well as pathological response rates after neoadjuvant treatment [24,25] but failed to improve overall survival in both early and advanced settings. Similar results have been obtained with other types of advanced cancer with improvements of progression-free survival with small or no clinical impact on overall survival [26,27,28,29].

The cancer stem cell theory has been largely developed in the last decade to explain resistance to cytotoxic agents [30]. Cancer stem cells display capacities of auto-renewal and differentiation and thus can drive resistance and disease recurrences [31,32,33].

Cancer stem cells have been described to display higher enzymatic activity of aldehyde dehydrogenase 1 (ALDH1) than differentiated cells, and can thus be identified using ALDH1 staining in various cancer localizations, including breast cancer [34,35,36,37,38]. Brain tumors models developed from cancer stem cells (CSC) synthetize higher levels of VEGF and can be inhibited with bevacizumab [39]. CSC secreting VEGF may also be localized in pro-angiogenic niches, with potentiation of antitumor effects when combining cytotoxic chemotherapy to antiangiogenic treatments [40,41]. Concerning breast cancer, in vitro models have suggested that CSCs express elevated levels of pro-angiogenic factors including VEGF, and that bevacizumab can decrease this pro-angiogenic effect [42].

As chemotherapy alone enriches breast CSC population after neoadjuvant treatment [30], we hypothesized that CSC inhibition by chemo-bevacizumab combinations can lead to a decrease or a stabilization of CSC rate within breast tumors. Differentiated cancer cells should indeed be killed by chemotherapy whereas CSC should be targeted by bevacizumab. We proposed to assess CSC inhibition by a chemotherapy–bevacizumab combination compared to chemotherapy alone for breast cancer patients treated in the neoadjuvant setting.

## 2. Experimental Section

### 2.1. Study Design

The AVASTEM trial (EudraCT Number: 2009-014773-40; ClinicalTrials.gov Identifier: NCT01190345) was conducted as an open-label, randomized (2:1), prospective, multicenter, phase II trial with chemotherapy with or without bevacizumab combination for the treatment of primary breast cancer candidate to receive preoperative chemotherapy. Its primary objective was to evaluate cancer stem cell (CSC) inhibition under treatment by assessing the percentage of residual ALDH1 positive cells after the fourth treatment cycle.

Secondary objectives were safety (as assessed according to CTCAE v3.0 criteria), pathological response (pCR) rate, disease-free survival (DFS), relapse-free survival (RFS), and overall survival (OS).

### 2.2. Patients

This trial was conducted in three French tertiary comprehensive cancer centers (Institut Paoli Calmettes, Marseille; Institut Curie, Paris; Institut Jean Godinot, Reims).

To be enrolled in this trial, patients had to have been diagnosed with a pathologically confirmed breast cancer, SBR grade 2 or 3 (except for inflammatory breast tumors), tumor size over 3 cm. Patients were 18 years or older; with a good performance status (ECOG ≤ 1); life expectancy of at least 3 months; adequate hematological, liver, and renal functions; without cardiac dysfunction (left ventricle ejection fraction ≥ 55%). Exclusion criteria were breast cancers of lobular subtype (except for HER2-positive, grade 3 or inflammatory breast tumors); prior treatment with bevacizumab; history of invasive cancer within the last 5 years (except skin carcinoma and in situ cervix cancers with appropriate treatment); uncontrolled high blood pressure (systolic > 150 mmHg and/or diastolic > 100 mmHg); history of congestive heart failure, unstable angina, myocardial infarction within the past 6 months; serious cardiac arrhythmia; thrombotic event within the last 6 months; history of coagulation disorder; history of digestive or non-digestive fistula or digestive perforation within the last 6 months; major surgery within the last 28 days; and history of anaphylaxis to monoclonal antibodies. All patients were affiliated to, or beneficiated to, a national insurance regimen. Patients were enrolled after signature of a written informed consent. All procedures were done in accordance with the 2008 Helsinki Declaration, after approval of the responsible ethics committees (institutional and national).

### 2.3. Treatments

Treatment scheme, dose reductions, and safety assessments were done according to protocol recommendations.

Patients randomized in arm A (experimental treatment) received four 21-day cycles of FEC100 IV infusions (5FU 500 mg/m^2^, epirubicin 100 mg/m^2^, and cyclophosphamide 500 mg/m^2^) on day 1 plus bevacizumab (15 mg/kg on day 1). They then received four 21-day cycles of docetaxel (100 mg/m^2^ on day 1) plus bevacizumab (15 mg/kg on day 1). Patients randomized in the control arm only received cytotoxic chemotherapy. Patients with HER2-positive tumors also received IV trastuzumab. No dose reduction was allowed for bevacizumab, and toxicities were managed according to EMA (European Medicines Agency) summary of product characteristics. Other drugs dose modifications were done in accordance to EMA guidelines.

### 2.4. Randomization Process

A stratified permuted block randomization method was used to allocate patients in a 2:1 ratio to receive FEC + bevacizumab or FEC alone. Block randomization lists were generated using a computer algorithm with randomly selected block sizes to ensure reproducibility and constant ratio of patients assigned between arms within blocks. The random allocation sequence and patients’ assignment were performed by the biostatics unit from the Institut Paoli-Calmettes. Clinicians from the three participating centers enrolled the patients.

### 2.5. Primary Objective Analysis

Breast CSC and progenitors can be identified by exploring the expression of an enzyme involved in retinoic acid metabolism: ALDH1 [34,43]. CSC inhibition was assessed in the intention to treat population as the percentage of ALDH1-positive cells identified using immunohistochemistry (IHC) as previously described with the ALDH1 antibody (Becton Dickinson biosciences) used at a 1/50 dilution [34], both before treatment and after the fourth treatment cycle. All patients with ADLH1 assessment prior to and during treatment were included in primary objective analysis.

### 2.6. Analysis of Safety Data

Safety assessments consisted of recording all adverse events (AEs) (CTCAE v3.0 criteria and NYHA for cardiac AEs) and serious adverse events (SAEs) collected during the treatment period and within 28 days after treatment completion. Cardiac AEs were collected for 12 months after treatment discontinuation. The safety population was defined as the subjects who received at least a partial dose of treatment.

### 2.7. Analysis of Clinical Efficacy Data

The efficacy population was defined as the group of subjects who received at least one chemotherapy cycle. Pathological complete response after treatment was planned to be defined according to Sataloff classification [44]. However, as most of recent literature data are mainly based on the more consensual ypT0/is pN0 definition [45], we described both classifications. DFS was defined as the time from treatment initiation to disease recurrence wherever the localization, secondary cancer diagnosis, or death, whatever occurred first. RFS was defined as the time from treatment initiation to disease recurrence or death. OS was defined as the time from treatment initiation to death, whatever the cause.

### 2.8. Statistical Analysis

The number of subjects to be included in this trial was 75 with a 2:1 ratio (50 patients in the experimental arm) to show that the increase of the percentage of ADLH1-positive cells was not upper than 5% after the fourth treatment cycle in the experimental arm, with an 80% power and an alpha-risk of 5%. An exact Wilcoxon rank-signed test was performed to confirm the rejection of the hypothesis that ALDH1+ cells rate increase was upper than 5% after four chemotherapy cycles in the experimental arm. *p*-Values, estimation of location parameters (Hodges Lehmann pseudo-median) and their one sided 95% confidence intervals (CI) were determined using exactRankTests v0.8-29 R-package [46].

Descriptive statistics were used to summarize the frequency, severity, duration, and relationship to treatment for all AEs occurring after the initiation of treatment. Categorical variables were described using counts and frequencies, and quantitative variables were described using medians and ranges. Patients’ characteristics were compared using exact Fisher’s tests for qualitative variables and rank-Wilcoxon’s tests for quantitative variables. Hazard ratios are provided with their bilateral confidence interval and Wald’s test *p*-value for significance. Follow-ups were estimated using the inverse Kaplan–Meier method. Patients lost to follow-up or without event were censored at the date of last news. Survival curves were estimated using the Kaplan–Meier method, and the median DFS, RFS, and OS were calculated with their 95% confidence intervals. Both univariate and multivariate analyses were conducted using Cox’s proportional hazard regression models including treatment arm, ADLH1 status at inclusion and pCR status as categorical explanatory variables. For Cox multivariate analysis, all the delays were considered from surgery date instead of inclusion due to an adjustment on pCR status (evaluated at surgery).

Except for the primary endpoint, all tests were two-sided. The level of statistical significance was set at α = 0.05. Statistical analyses were carried out with the SAS^®^ software version 9.3 (SAS Institute Inc., Cary, NC, USA) and the R software version 3.0.3 (https://www.R-project.org/). This study was performed according to the CONSORT statements [47], (see Supplementary checklist).

## 3. Results

### 3.1. Population Description 

**Figure 1:**
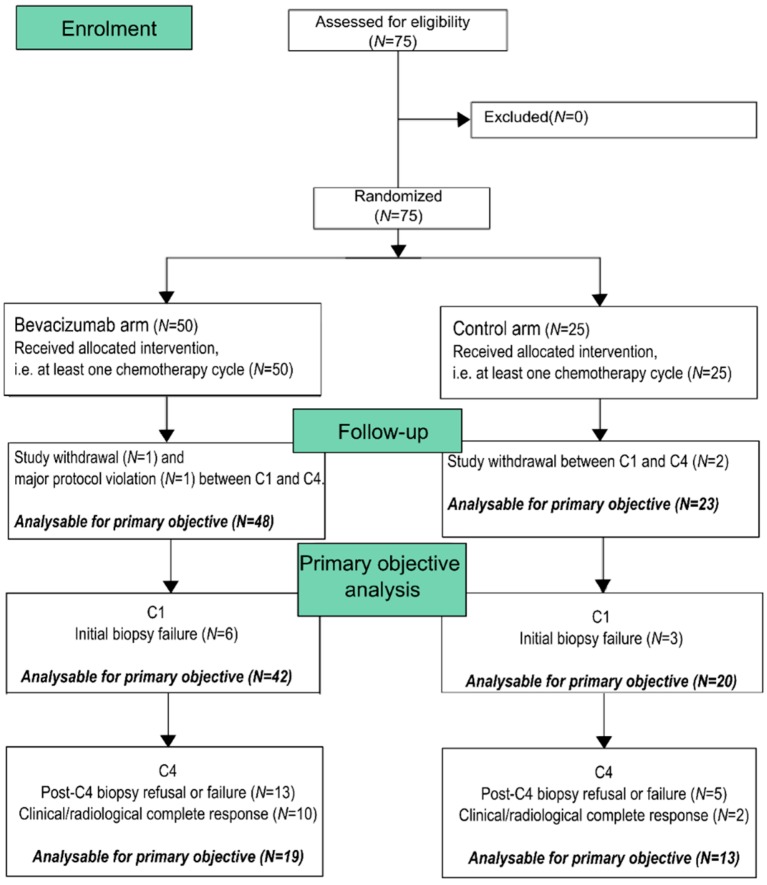
CONSORT flow diagram.

Seventy-five patients were included from March 2010 to July 2012 (Figure 1). Follow-up was stopped five years after inclusion of the last patients, i.e., in October 2017. Fifty patients received the experimental treatment (arm A) and twenty-five were randomized in the control arm (arm B). There was no imbalance between arms (Table 1). Median age at inclusion was 50.0 years for arm A versus 49.8 for arm B. Ten patients had de novo metastatic breast cancer (arm A = 14%, arm B = 12%). Out of non-metastatic cases, most of them were larger than 5cm (T3: 37.2% vs. 31.8%; T4a–b: 2.3% vs. 9.1%; T4d: 18.6% vs. 13.6%). There was a trend in higher rates of lymph node involvement for non-metastatic patients included in arm B (N1: 52.4% vs. 77.3%; N2: 16.7% vs. 4.5%; N3: 2.4% vs. 9.1%; *p* = 0.06). Pathologically-defined molecular subtypes were well balanced: fourteen cases (28%) were HER2-positive in arm A (including 6 HR-positive and 8 HR-negative tumors) versus eight (32%) in arm B (including 3 HR-positive and 5 HR-negative tumors). Triple negative tumors rates were also similar for both arms (42.9% vs. 40%).

### 3.2. Treatment Administration and Safety

More than 80% of patients received eight chemotherapy cycles (A: 88%, B: 84%). Only five patients did not receive the four scheduled cycles of FEC. Seven patients (arm A) and 4 patients (arm B) did not receive all the planned docetaxel infusions. All the patients in the experimental arm received at least one cycle of bevacizumab. Out of them 43 (86%) received at least four cycles and 28 (56%) received the eight planned bevacizumab courses.

Concerning safety (Table 2), 94% of patients included in the experimental arm and 88% in the control arm experimented grade 3 to 4 adverse events. The most frequent were haematological disorders with 46 to 48% of grade 3 to 4 neutropenia, with 34% of febrile neutropenia in arm A and 8% in arm B. High grades of anaemia, asthenia, nausea, mucitis, and cutaneous disorders were also more frequent in the experimental arm (18% vs. 4%, 14% vs. 4%, 10% vs. 0%, 22% vs. 8%, and 20% vs. 12%, respectively). Regarding bevacizumab-related adverse events, severe high blood pressure increase was observed for two patients (4%) in arm A (vs. 0 in arm B); three cases of thrombotic event were observed for patients in arm A (none in the control arm).

No treatment-related death was reported during study duration.

### 3.3. Primary Endpoint: Percentage of ADLH1+ Cells

**Figure 2:**
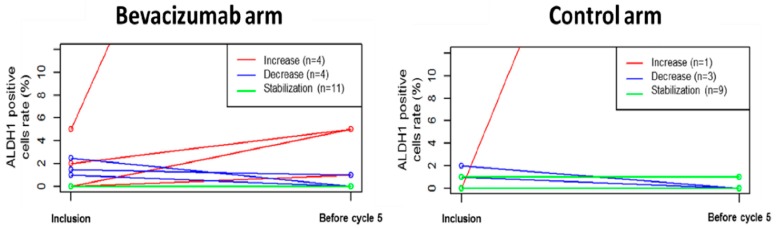
ADLH1 positive cells rates modifications after the first four cycles of treatment (each line represents one patient). Red lines represent patients presenting an increase of ALDH1+ cells rate. Blue lines represent patients presenting a decrease of ALDH1+ cells rate. Green lines represent patients without ALDH1+ cells rate modification.

IHC analysis was performed to identify tumors with ALDH1 expression at inclusion. We evaluated the percentage of ALDH1+ cells for each sample at inclusion and after the fourth chemotherapy cycle. Absence of a large increase of ALDH1 expression was explored as a surrogate marker of CSC inhibition due to chemotherapy-bevacizumab combination. ALDH1 data before and after four chemotherapy cycles were available for 19 patients in arm A and 13 patients in arm B. The primary endpoint was thus not evaluable in 43 patients, of whom 12 patients were excluded from the analysis set due to complete remission at the end of the fourth cycle of chemotherapy (Figure 1). Most of the patients randomized in the experimental arm with both biopsy analyses available experimented a low increase (<5%) or a decrease of ALDH1+ cells rate (pseudo-median = −0.125, one-sided 95% CI (−∞–0), *p* = 0.001, Wilcoxon test, *N* = 19), (Figure 2). ALDH1 expression was also significantly not higher after chemotherapy in the control arm (pseudo-median = −0.25, one-sided 95% CI (−∞–0), *p* = 0.006, *N* = 13).

We then exploratory assessed ALDH1 expression levels on post-chemotherapy (after the 8th cycle) samples. ALDH1 pseudo-median of difference between post-chemo and pre-chemo samples was −0.125 (one-sided 95% CI (−∞–0), *p* < 0.001, *N* = 19) in the experimental arm versus −0.25 (one-sided 95% CI (−∞–10), *p* = 0.14, *N* = 7) for the chemotherapy only arm. When adding cases with complete pathological response after chemotherapy to patients without high ALDH1+ rate increase, we observed that these results were similar both for the experimental arm (pseudo-median = −0.125, one-sided 95% CI (−∞–0), *p* < 0.001, *N* = 37) and the control arm (pseudo-median = −0.25, one-sided 95% CI (−∞–0), *p* = 0.016, *N* = 11).

### 3.4. Efficacy

#### 3.4.1. Efficacy for the Whole Population

Regarding pathological response after chemotherapy (Table 3), a not statistically significant difference according to treatment arm was observed when using Sataloff classification (51.1% vs. 31.8%; OR = 2.24, 95% CI (0.77–6.54), *p* = 0.14). When using the usual definition of pathological complete response, there was a trend for pCR rate improvement for patients who received bevacizumab (42.6% vs. 18.2%; OR = 3.33, 95% CI (0.98–11.38), *p* = 0.06).

**Figure 3:**
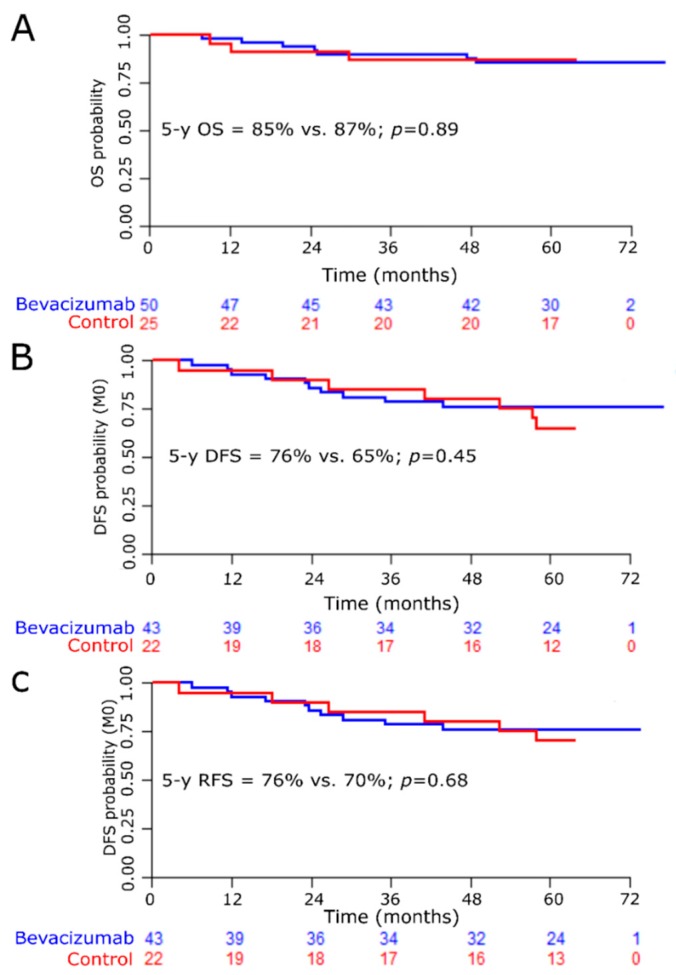
Kaplan–Meier curves. (**A**): Overall survival for the whole cohort. (**B**): Disease-free survival for non-metastatic patients. (**C**): Relapse-free survival for non-metastatic patients.

Median follow-up was 60.9 months for the whole population (95% CI (60.4–61.8)) with no difference according to treatment arm (61.3 months, 95% CI (60.2–62.5), for arm A vs. 60.6 months, 95% CI (60.2–61.6), for arm B). The 5-y OS was 86% (95% CI (75–92)) for the whole cohort and 90% (95% CI (80–96)) for non-metastatic patients. There was no OS difference according to treatment arm (5-y OS = 85% for arm A vs. 87% for arm B; *p* = 0.89; HR = 1.10, 95% CI (0.28–4.25)), (Figure 3A). Considering non-metastatic patients, 5-y DFS was 73% (95% CI (60–82)) for the whole population, without improvement for patients who received bevacizumab (5-y DFS = 76% for arm A vs. 65% for arm B; *p* = 0.45; HR = 0.69, 95% CI (0.26–1.81)), (Figure 3B). Results were similar regarding RFS. Five-y RFS was 76% for arm A vs. 70% for arm B (*p* = 0.68; HR = 0.81, 95% CI (0.29–2.23)), (Figure 3C).

#### 3.4.2. Efficacy according to ALDH1 Status

At inclusion, 21 of 63 patients with analyzable samples were ADLH1+ (33% in both treatment arms). ALDH1 status was also available for 32 patients after the fourth chemotherapy cycle. Of them, twenty-two percent had ADLH1+ tumors. Among the six ALDH1+ cases at inclusion in arm A, three were still ADLH1+ after four chemotherapy cycles (vs. three of four in arm B). Two of 13 ADLH1− cases became ADLH1+ in arm A versus one of nine in arm B. ADLH1 status at inclusion was not correlated to pCR (ypT0/is pN0 definition) after neoadjuvant treatment: OR (ALDH1− vs. ADLH1+) = 2.74, 95% CI (0.84–8.97), *p* = 0.10). Similar results were observed for OS (HR = 0.69, 95% CI = (0.15–3.06), *p* = 0.62), DFS (HR = 0.94, 95% CI = (0.37–2.40), *p* = 0.90), and RFS (HR = 1.10, 95% CI = (0.41–2.94), *p* = 0.85). Multivariate Cox regression analyses including ALDH1 status at inclusion, treatment arm, and pCR status failed to show survival benefits for ADLH1-positive tumors (Tables S1–S3).

## 4. Discussion

This randomized prospective phase II trial aimed to bring evidences of CSCs inhibition using a chemotherapy-bevacizumab combination. As hypothesized, we observed that ADLH1+ cells rate was not increased above the predefined threshold for patients receiving bevacizumab-based neoadjuvant chemotherapy. However, as similar results were obtained for the control group, an impact of angiogenesis inhibition on the CSC population cannot be confirmed.

Contrarily to our initial hypothesis, some preclinical results published after initiation of our trial intriguingly suggested that hypoxia generated by antiangiogenic agents, including bevacizumab, could stimulate the population of CSCs and contribute to the resistance of these drugs and the lack of survival benefit provided by these compounds in breast cancer [48]. An increase in CSCs was particularly noticeable when treatment was delivered in the context of large and established tumors, a setting comparable to that of neoadjuvant treatment, most likely in relation to a greater overall volume for hypoxia to occur. Nevertheless, these preclinical observations were not clearly validated in vivo, and we did not observe CSCs stimulation during bevacizumab treatment in the present trial.

The ADLH1 status at inclusion, which can be seen as a surrogate of the CSC population, was also neither prognostic nor predictive of pCR after treatment. These results are contradictory to other published data. In a Japanese cohort of more than 100 breast cancers receiving preoperative chemotherapy, ALDH1-positive cases were associated with a low pCR rate (9.5% vs. 32.2%; *p* = 0.037). Analyses done before and after neoadjuvant chemotherapy showed an increase of the proportion of ALDH1-positive tumor cells for patients who did not achieve pCR [49]. These results were confirmed in another set of 119 patients receiving anthracyclines and taxanes before breast surgery, for whom ALDH1 negativity at baseline was significantly associated with pCR, and ALDH1 expression was increased after chemotherapy [50]. In this study, ALDH1 expression in the residual tumor was also prognostic, with worse overall survival for ALDH1-positive cases. Similar results were observed in a retrospective work including more than 650 breast cancer patients [51]. We did not find such a correlation between ALDH1 expression at diagnosis and treatment efficacy in our study. Concerning pCR, the results were inconclusive (*p* = 0.10) probably because of a lack of power, and this trial was indeed not powered for pCR nor for survival analyses.

Pathological response rates were surprisingly high in our set with, according to the classification used, 42 to 51% of pCR in the bevacizumab arm and 18 to 32% in the control arm. These rates decreased our capacity to obtain post-treatment tumor samples for ALDH1 expression analysis. However, even though high, these rates are in accordance to the data published during the last years for the tumor phenotypes described in this set. Nearly 60% of patients included harbored HR-negative tumors, including more than 40% with triple-negative cancer. Triple negative breast cancer is now known to display higher rates of pCR, and our results are in accordance with what has been previously published with pCR rates ranging from 25% to 60% [51,52,53].

In the GeparQuinto trial, exploring the addition of VEGF-R inhibition to anthracycline and taxane-based neoadjuvant therapy, pCR rates in the TNBC subset were significantly higher with bevacizumab (39.3% vs. 27.9%, *p* = 0.003) [53], with results close to what we observed in our cohort. However, as we also notified, survival was not improved with the combination for TNBC (HR = 0.99 for DFS, *p* = 0.94; HR = 1.02 for OS, *p* = 0.89) nor for the whole HER2-negative cohort, (HR = 1.03 for DFS, *p*= 0.78; HR = 0.97 for OS, *p* = 0.84) [54]. To avoid this issue of TNBC excess leading to high pCR rates in both groups, we should have decreased the number of TNBC patients included. However, this would have led to insufficient enrolment within the predefined inclusion period.

This trial has some limits which deserve to be underlined. Firstly, the definition of the main endpoint was of importance at time of trial design but could be more criticized if this study takes place in 2019. Improvement of the surrogate markers of CSCs could have allowed us to reach our primary objective. Additional staining focused on the ALDH1A1 isoform, or other stem cells markers such as CD44+/CD24− may be of interest [55,56]. However recent studies suggest that CD44+ CD24− phenotype and ALDH1 phenotype may form part of two interconvert dynamic EMT states. Mesenchymal-like CSCs (CD44+/CD24− are more quiescent whereas epithelial-like BCSCs (ALDH1+) are more proliferative [57]. In the neoadjuvant setting, ALDH1 staining only displayed a prognostic value for patients in terms of pCR or metastasis-free survival [58]. We thus chose to monitor CSC during neoadjuvant chemotherapy using ALDH1 staining. The ALDEFLUOR assay, based on the assessment of ALDH enzymatic activity, could also have been evaluated, but is very unlikely to be developed in routine practice as it needs early processing of fresh tumor samples and flow cytometry experiments [55]. Secondly, even though the randomization was of importance to look for an association between bevacizumab treatment and CSC enrichment as suggested by Li et al. [30], the lack of blind could have had an impact on efficacy data, including our primary endpoint. Thirdly, CSCs may not be a good candidate to predict neoadjuvant treatments efficacy. We have recently described that pCR rates after chemotherapy and bevacizumab-based neoadjuvant treatments in inflammatory breast cancer are correlated to circulating tumor cells (CTC) detection [59]. CTC-positive cases displayed worse three-year DFS (39% vs. 70%) and OS. CTC status at baseline was an independent prognostic feature in multivariate analysis. A team from Harvard has also recently reported that another type of tumor cells with (but not only with) CSC properties, called AKT1^low^ quiescent cancer cells, seem to be resistant to chemotherapy in the neoadjuvant setting for triple negative breast cancer [60]. Concerning the current study, as it was designed as a proof of concept trial with a biological primary endpoint, its sample-size was limited, response and survival endpoints were secondary, and definitive conclusions related to bevacizumab contribution to treatment efficacy can only be considered as preliminary. However, larger and more robust results have been published in randomized trials evaluating adjuvant bevacizumab efficacy for early breast cancers [61]. These data are in accordance with our results with no survival gain with bevacizumab-based combinations in the (neo)adjuvant setting, despite higher rates of pathological response [24,25,54,62]. Similar results have been obtained in the early setting for other tumor localizations, with no disease-free survival or overall survival improvements [63,64]. Even though pCR has been widely described to be prognostic in early breast cancer, survival gains secondary to treatment-induced pCR improvement is still debated. A large meta-analysis published a few years ago thus showed that trials describing pCR gains did not result in survival benefits, with the exception of the NOAH trial which introduced trastuzumab in the preoperative systemic regimen for HER2-positive cases [45].

## 5. Conclusions

Bevacizumab impact on CSC was not higher than that of chemotherapy alone for the cases enrolled in this trial. Moreover, despite that bevacizumab-chemotherapy combination enhanced pCR rates compared to chemotherapy alone for breast cancer receiving neoadjuvant chemotherapy, it did not improve survival. These observations warrant to be deeply explored in larger series and for other tumor localizations for which bevacizumab is more widely used such as ovarian, colorectal, and cervical cancers [27,28,65,66,67].

## Figures and Tables

**Table 1:** Clinical and pathological features at inclusion. When required, results are notified as *N* (% of cases with data available).

		Bevacizumab Arm (*N* = 50)	Control Arm (*N* = 25)	*p*-Value
**Clinical features**				
Age at inclusion, years (median, min–max)		50.0 (24.3–66.9)	49.8 (28.4–68.5)	0.47
Menopausal		9 (18)	6 (24)	0.55
Tumor size	T2	21 (42)	10 (40)	1.00
	T3	17 (34)	9 (36)	
	T4	12 (24)	6 (24)	
Axillary lymph node positive		37 (75.5)	22 (88)	0.24
Metastatic disease at diagnosis		7 (14)	3 (12)	1
**Pathological features at diagnosis**				
HR status	Positive	21 (42)	10 (40)	1
	Negative	29 (58)	15 (60)	
HER2 status	Positive	14 (28)	8 (32)	0.86
	Negative	35 (70)	17 (68)	
	Equivocal	1 (2)		
Triple negative phenotype		21 (42.9)	10 (40)	1
SBR grade	1–2	12 (24)	9 (36)	0.41
	3	37 (76)	16 (64)	
Lymphovascular invasion positive		6 (13)	1 (4)	0.41

**Table 2:** Most common adverse events (Common Terminology Criteria for Adverse Events version 3.0). Only adverse events (AEs) related to treatment and with an incidence ≥ 10% for all grades or ≥ 5% for grade ≥ 3 are presented.

	Bevacizumab Arm				Control Arm			
	All Grades		Grade ≥ 3		All Grades		Grade ≥ 3	
**Adverse events**	*N*	%	*N*	%	*N*	%	*N*	%
**Haematological**								
Anaemia	13	26	9	18	5	20	1	4
Lymphopenia	9	18	9	18	6	24	6	24
Neutropenia	24	48	23	46	12	48	12	48
Febrile neutropenia	17	34	17	34	2	8	2	8
**Non-haematological**								
Asthenia	37	74	7	14	18	72	1	4
Anorexia	9	18	2	4	4	16	1	4
Weight lost	13	26	0	0	1	4	0	0
Constipation	16	32	0	0	7	28	0	0
Diarrhoea	17	34	0	0	6	24	0	0
Nausea	43	86	5	10	20	80	0	0
Vomiting	18	36	3	6	2	8	0	0
Abdominal pain	7	14	1	2	2	8	0	0
Stomach pain	10	20	0	0	6	24	0	0
Dysphagia	8	16	0	0	1	4	0	0
Mucitis	39	78	11	22	17	68	2	8
Epistaxis	30	60	0	0	1	4	0	0
Arthralgia	10	20	1	2	11	44	1	4
Myalgia	18	36	0	0	12	48	0	0
Peripheral neuropathy	6	12	0	0	3	12	0	0
Cutaneous toxicities	43	86	10	20	21	84	3	12
Amenorrhea	10	20	5	10	9	36	6	24
High blood pressure	14	28	2	4	2	8	0	0
Headaches	15	30	0	0	1	4	0	0

**Table 3:** Pathological response rates. Results are notified as *N* (% of cases with data available).

		Bevacizumab Arm (*N* = 50)	Control Arm (*N* = 25)	Odds Ratio (95% CI)	*p*-Value
**Sataloff classification**	pCR	23 (51.1)	7 (31.8)	2.24 (0.77–6.54)	0.14
	Non-pCR	22 (48.9)	15 (68.2)		
	Missing data	5	3		
**ypT0/is pN0 definition**	pCR	20 (42.6)	4 (18.2)	3.333 (0.98–11.38)	0.06
	RD	27 (57.5)	18 (81.8)		
	Missing data	3	3		

pCR: pathological complete response, RD: invasive residual disease in breast or lymph nodes.

## Supplementary Materials

The following are available online at https://www.mdpi.com/2077-0383/8/5/612/s1, Table S1: Overall survival for the whole population, Table S2: Disease-free survival for M0 patients, Table S3: Relapse-free survival for M0 patients.
