# Supplementary material for: Stem Cells Inhibition by Bevacizumab in Combination with Neoadjuvant Chemotherapy for Breast Cancer
Source: J Clin Med. 2019 May 6;8(5):612. doi: 10.3390/jcm8050612 (PMC6572380; doi:10.3390/jcm8050612)
Supplement: Supplementary file 1 [file jcm-08-00612-s001.zip › AVASTEM-IPC 2009-001_PRO.pdf]

Institut Paoli-Calmettes

Centre régional de lutte contre le cancer Provence-Alpes-Côte d'Azur

Essai de phase II de preuve du concept : Evaluation de l'activité anti-cellules souches cancéreuses du bevacizumab administré en combinaison à la chimiothérapie néoadjuvante dans les cancers du sein

AVASTEM / IPC 2009-001

N° EudraCT : 2009-014773-40

Version n° 4 du 24/06/11

Investigateur coordonateur :

Dr Jean-Marc Extra
Institut Paoli-Calmettes
Service d'Oncologie Médicale
232, Bd Sainte Marguerite - BP 156
13273 Marseille Cedex 9
Tél. : 04 91 22 33 02 - Fax : 04 91 22 35 52
E-mail : extrajm@marseille.fnclcc.fr

Promoteur :

Bureau d'Etudes Cliniques
Institut Paoli-Calmettes
232, Bd Sainte Marguerite - BP 156
13273 Marseille Cedex 9
Tél. : 04 91 22 37 78 - Fax : 04 91 22 36 01
E-mail : bec@marseille.fnclcc.fr

Projet financé par un PHRC 2009

Toute reproduction partielle ou totale du présent document est interdite sans autorisation de l'IPC
© IPC- janvier 2008

1 - APPROBATION ET SIGNATAIRES DU PROTOCOLE

Titre : Essai de phase II de preuve du concept : Evaluation de l'activité anti-cellules souches cancéreuses du bevacizumab administré en combinaison à la chimiothérapie néoadjuvante dans les cancers du sein – AVASTEM / IPC 2009-001

AUTORITE COMPETENTE	Agence Française de Sécurité Sanitaire des Produits de Santé (Afssaps)	Date d'autorisation : 29/10/09
		Réf. Afssaps : A91213-45
COMITE DE PROTECTION DES PERSONNES	Nom du CPP :<br>Comité de Protection des Personnes Sud Méditerranée I	Date d'avis : 04/01/10
		Réf. CPP : 09 45

COMITE DE REDACTION	Dr Jean-Marc EXTRA<br>Dr Anthony GONÇALVES<br>Dr Emmanuelle CHARAFE-JAUFFRET

NOMS ET TITRES DES RESPONSABLES	COORDONNÉES	DATE (jj-mm-aa)	SIGNATURE
Responsable du BEC<br>**Dr Dominique GENRE**	Bureau d'Etudes Cliniques<br>Institut Paoli Calmettes<br>232 bd Sainte Marguerite – BP 156<br>13273 Marseille Cedex 9<br>Tél : 04 91 22 37 78<br>Fax : 04 91 22 36 01<br>Email : bec@marseille.fnclcc.fr		
Chef de Projet<br>**Dr Agnès BOYER CHAMMARD**	Bureau d'Etudes Cliniques<br>Institut Paoli Calmettes<br>232 bd Sainte Marguerite – BP 156<br>13273 Marseille Cedex 9<br>Tél : 04 91 22 37 78<br>Fax : 04 91 22 36 01<br>Email : bec@marseille.fnclcc.fr		
Investigateur Coordonnateur<br>**Dr Jean-Marc EXTRA**	Département d'Oncologie<br>Institut Paoli Calmettes<br>232 bd Sainte Marguerite – BP 156<br>13273 Marseille Cedex 9<br>Tél : 04 91 22 33 02<br>Email : extrajm@marseille.fnclcc.fr		
Biostatisticiens<br>**M. Jean-Marie BOHER**<br>**M. Benjamin ESTERNI**	Bureau d'Etudes Cliniques<br>Institut Paoli Calmettes<br>232 bd Sainte Marguerite – BP 156<br>13273 Marseille Cedex 9<br>Tél : 04 91 22 37 78<br>Fax : 04 91 22 36 01<br>Email : bec@marseille.fnclcc.fr		

TABLE DES MATIÈRES

1 - APPROBATION ET SIGNATAIRES DU PROTOCOLE 2

2. INTRODUCTION ET RATIONNEL DE L'ETUDE 5

2.1 SYNTHÈSE DES RÉSULTATS ANTÉRIEURS 5
2.2 DONNÉES DES TRAITEMENTS À ÉVALUER 7
2.3 JUSTIFICATION DE L'ESSAI 11
2.4 PERSPECTIVES 12

3. OBJECTIFS DE LA RECHERCHE 13

3.1 OBJECTIF PRINCIPAL 13
3.2 OBJECTIFS SECONDAIRES 13

4. CONCEPTION DE LA RECHERCHE 13

4.1 CRITÈRE D'ÉVALUATION 13
4.2 MÉTHODOLOGIE ET PRÉSENTATION SCHÉMATIQUE 13
4.3 MESURES PRISES POUR ÉVITER OU RÉDUIRE LES BIAIS 14
4.4 POSOLOGIE, MODALITÉS D'ADMINISTRATION, CONDITIONNEMENT, ÉTIQUETAGE 14
4.5 DURÉE DE PARTICIPATION DES PERSONNES ET CHRONOLOGIE DES PÉRIODES DE L'ESSAI 15
4.6 DESCRIPTION DES RÈGLES D'ARRÊT DU TRAITEMENT 16

5. SELECTION DES PERSONNES DE LA RECHERCHE 16

5.1 CRITÈRES D'INCLUSION 16
5.2 CRITÈRES DE NON-INCLUSION 17
5.3 ENREGISTREMENT DES PATIENTS 18

6. TRAITEMENTS 18

6.1 DESCRIPTION DES TRAITEMENTS NÉCESSAIRES À LA RECHERCHE 18
6.2 TRAITEMENTS AUTORISÉS ET INTERDITS 18
6.3 CONDITIONS DE STOCKAGE, DISTRIBUTION 19
6.4 PROCÉDURE DE COMPTABILITÉ 19

7. CRITERES D'EVALUATION 19

7.1 DESCRIPTION DES PARAMÈTRES 19
7.2 MÉTHODES ET CALENDRIER PRÉVUS 21

8. EVALUATION DES EVENEMENTS INDESIRABLES GRAVES 23

8.1 DÉFINITION 23
8.2 DÉFINITION D'UN ÉVÉNEMENT INDÉSIRABLE GRAVE ATTENDU (EIG-A) 23
8.3 DÉFINITION D'UN ÉVÉNEMENT INDÉSIRABLE GRAVE INATTENDU (EIG-I) 23
8.4 CRITÈRE D'INTENSITÉ 23
8.5 PÉRIODE DE RECUEIL DES ÉVÈNEMENTS INDÉSIRABLES GRAVES 24
8.6 CONDUITE À TENIR EN CAS D'ÉVÉNEMENT INDÉSIRABLE GRAVE 24

9. STATISTIQUES 24

9.1 ANALYSE STATISTIQUE DES DONNÉES 24
9.2 NOMBRE DE PATIENTS ET DURÉE DE L'ÉTUDE 25
9.3 DEGRÉ DE SIGNIFICATION STATISTIQUE PRÉVU 25
9.4 CRITÈRES STATISTIQUES D'ARRÊT DE LA RECHERCHE 25
9.5 MÉTHODE DE PRISE EN COMPTE DES DONNÉES MANQUANTES 25
9.6 GESTION DES MODIFICATIONS APPORTÉES AU PLAN D'ANALYSE DE LA STRATÉGIE INITIALE 25
9.7 CHOIX DES PERSONNES À INCLURE DANS LES ANALYSES 25

10. DROITS D'ACCÈS AUX DONNÉES 25

11. CONTROLE ET ASSURANCE QUALITE 26

12. CONSIDERATIONS ETHIQUES ET REGLEMENTAIRES 26

12.1 COMITÉ DE PROTECTION DES PERSONNES (CPP) 26
12.2 DÉCLARATION AUX AUTORITÉS COMPÉTENTES 26
12.3 INFORMATION ET CONSENTEMENT DES PARTICIPANTS 26
12.4 RESPONSABILITÉS DES INVESTIGATEURS 27
12.5 RESPONSABILITÉS DU PROMOTEUR 27
12.6 COMITÉ DE PATIENTS 28

13. TRAITEMENT DES DONNÉES ET CONSERVATION DES DOCUMENTS ET DES DONNÉES RELATIVES À LA RECHERCHE 28

14. FINANCEMENT ET ASSURANCE 28

15. RÈGLES RELATIVES À LA PUBLICATION 28

REFERENCES 30

ANNEXES 35

ANNEXE 1 : TABLEAU RECAPITULATIF DES INVESTIGATIONS 36
ANNEXE 2 : EVALUATION DE L'ETAT GENERAL EN FONCTION DE LA CLASSIFICATION DE KARNOFSKY (OMS) 37
ANNEXE 3 : MACROBIOPSIES ET MARQUAGE ALDH1 EN IMMUNOHISTOCHIMIE 38
ANNEXE 4 : ETUDES ANCILLAIRES 40
ANNEXE 5 : GESTION DES TOXICITÉS INDUITES PAR LE BEVACIZUMAB 47
ANNEXE 6 : ECHELLE DE TOXICITE NCI CTC-AE V3.0 52
ANNEXE 7 : CLASSIFICATION DE LA NYHA 53
ANNEXE 8 : RÉSUMÉ DES CARACTÉRISTIQUES DES PRODUITS 54

2. INTRODUCTION ET RATIONNEL DE L'ETUDE

2.1 Synthèse des résultats antérieurs

2.1.1 Cancer du sein et cellules souches cancéreuses (CSC)

Bien que le concept de cellules souches cancéreuses soit un vieux concept, ce n'est que récemment que les progrès dans la biologie des cellules souches ont mis au point des modèles expérimentaux permettant de l'objectiver[1]. Selon ce modèle, les tumeurs prendraient leur origine dans les cellules souches ou dans les progéniteurs précoces des tissus par une dérégulation des mécanismes d'autorenouvellement. L'autorenouvellement est le processus par lequel les cellules souches donnent naissance à des cellules identiques à elles-mêmes. Dans les organes adultes, ce processus est à la base du maintien et de la réparation tissulaire après un dommage. Le pendant de l'autorenouvellement dans l'organogenèse et l'homéostasie tissulaire est la différenciation cellulaire. Les cellules souches se différencient et génèrent des progéniteurs multipotents qui donnent naissance à leur tour à des progéniteurs plus tardifs et à des cellules différenciées matures qui assurent la fonctionnalité de l'organe considéré. Du fait de leurs capacités d'autorenouvellement, les cellules souches possèdent un potentiel prolifératif élevé, qui en plus de leur longue durée de vie, en font des candidats idéals pour accumuler des mutations tout au long de leur vie, et se transformer le cas échéant. Les cellules couches cancéreuses possèdent les mêmes propriétés que les cellules souches normales : elles sont capables de s'autorenouveler, et donc peuvent diriger tumorigénicité, récidive et métastase. Elles peuvent aussi se différencier, même s'il s'agit d'une «différenciation aberrante» dans le cadre du cancer et sont capables de générer la masse hétérogène des cellules qui constituent la tumeur. Ces cellules tumorales différenciées constituent la masse tumorale mais sont incapables de donner naissance à une tumeur car leur potentiel prolifératif est limité (dans le temps) et qu'elles ne peuvent s'autorenouveler. Les cellules souches cancéreuses peuvent provenir de la transformation oncogénique d'une cellule souche normale ou d'un progéniteur qui aurait acquis des capacités d'autorenouvellement.

Les arguments prouvant le concept de cellules souches cancéreuses se sont accumulés rapidement ces dernières années en partie grâce au développement de modèles murins qui permettent de tester cette hypothèse de façon expérimentale. En 1997, le groupe de John Dick démontrait que les leucémies humaines sont générées à partir d'une petite population de cellules souches leucémiques ayant la capacité de transférer cette activité leucémogène dans des modèles NOD/SCID[2]. Ce concept s'est ensuite étendu aux tumeurs solides grâce aux travaux de Clarke et Wicha qui ont montré que les cancers du sein comportaient une population caractérisée par l'expression des marqueurs de surface CD44+ CD24- lin-. Deux cents cellules ayant ce phénotype sont capables de générer un cancer du sein humain dans le modèle murin NOD/SCID. De plus en accord avec le modèle de cellules souches, les tumeurs générées récapitulent l'hétérogénéité de la tumeur initale[3]. Le même groupe démontrait plus récemment que l'activité enzymatique de l'aldéhyde déshydrogénase pouvait être utilisée pour isoler une sous population cellulaire à partir de sein normal ou de cancer du sein qui possédait des capacités de cellule souches. La population ALDEFLUOR-positive isolée à partir de mammoplastie de réduction est capable de reconstituer des structures ductulo-alvéolaires normales dans des modèles de xénogreffes dans la glande mammaire de souris NOD/SCID préalablement humanisée[4]. De plus, les cellules ALDEFLUOR-positives isolées de cancers du sein ou de lignées cellulaires mammaires ont aussi des propriétés de cellules souches comme le montre leur capacité à reconstituer des tumeurs à travers des passages en série ainsi que leur potentiel à générer la diversité phénotypique de la tumeur initiale (Charafe-Jauffret Cancer Res in press).
Dans le sein comme dans d'autres organes, il existe un niveau de preuve de plus en plus élevé que le développement et la croissance des cancers est géré par des cellules souches

cancéreuses initiatrices du cancer qui peuvent contribuer aux métastases tumorales et à la résistance thérapeutique[2,5-8].

2.1.2 Détection du contingent CSC et cancer du sein

Les cellules souches du cancer du sein ont été historiquement caractérisées sur le phénotype CD44+/CD24-/lin- capable de transplantation sériée dans les souris NOD/SCID[3]. Depuis toujours dans le groupe de M Wicha à l'Université du Michigan (MI, USA), l'activité enzymatique d'une enzyme du métabolisme de l'acide rétinoïque, l'aldéhyde déshydrogénase, a été utilisée pour isoler des cellules ayant des propriétés de cellules souches à partir de sein normal humain. En utilisant la cytométrie de flux et le test ALDEFLUOR qui mesure cette activité enzymatique, Wicha et collaborateurs ont aussi montré qu'ils pouvaient isoler une population de cellules souches cancéreuses à partir de tumeurs primaires mammaires humaines ayant poussé en xénogreffe en souris NOD/SCID. De plus, ils ont montré que la détection in situ de la protéine ALDH1 par immunomarquage sur tissus fixé et inclus en paraffine permettait de détecter des cellules souches normales et tumorales[4]. Notre institution a développé par le biais du Dr Charafe-Jauffret une collaboration étroite avec l'équipe de Max Wicha et de nouvelles données confirmant l'intérêt de l'utilisation d'ALDH dans le cancer du sein sont sur le point d'être publiées. En effet, le même marqueur a été utilisé pour isoler une sous population ayant des capacités de cellules souches cancéreuses à partir de lignées cellulaires capables d'auto renouvellement, de différenciation ainsi que d'initier des tumeurs (Charafe-Jauffret et al, Cancer Research in press)

2.1.3 Angiogénèse, VEGF et cancer du sein

La néoangiogénèse, terme désignant le développement de nouveaux capillaires à partir de vaisseaux préexistants, est un processus indispensable lors du développement tumoral[9] . Le processus de néoangiogénèse est étroitement régulé grâce à un équilibre entre facteurs locaux pro- et antiangiogéniques. La perturbation de l'équilibre entre facteurs pro et antiangiogéniques connue sous le terme de «switch angiogénique» est nécessaire à la croissance tumorale et à ses besoins en oxygène et nutriments au-delà d'un volume de 1 mm^3.

Le Vascular endothelial growth factor (VEGF) est le plus puissant et le plus spécifique des facteurs promoteurs de l'angiogénèse décrit ; il est un élément régulateur clef de la formation de néovaisseaux pendant l'embryogénèse, la croissance squelettique ou encore la physiologie de l'appareil génital féminin. Il est également et surtout impliqué dans l'angiogénèse pathologique telle que celle associée à la croissance tumorale. Le VEGF-A, qui généralement est désigné comme le VEGF, est le chef de file d'une famille de molécules homologues, 6 d'entre elles ayant été identifiées incluant VEGF-B, VEGF-C, VEGF-D, VEGF-E et PIGF. Les protéines de la famille du VEGF sont à l'origine de nombreux effets biologiques incluant la division et la migration des cellules endothéliales, l'induction de la dégradation et du remodelage protéolytique de la matrice extracellulaire, l'augmentation de la perméabilité vasculaire et la survie des vaisseaux néoformés[10 11].

Les cellules tumorales stimulent les cellules endothéliales quiescentes et induisent leur prolifération et la formation des néovaisseaux par l'intermédiaire de la fixation du VEGF sur ses récepteurs spécifiques endothéliaux initiant une cascade de signalisation intracellulaire comprenant notamment des voies de prolifération, de migration et de survie. Au moins deux récepteurs tyrosine kinase de haute affinité sont engagés par le VEGF, flk-1/KDR (VEGFR2) et à un moindre degré flt-1 (VEGFR1)[12]. Les néovaisseaux sont extrêmement dépendants du niveau d'expression du VEGF qui apparaît up-régulé dans nombre de tumeurs[10,13,14] et associé à un mauvais pronostic[9,11,14-20] ainsi qu'à une relative résistance à la chimiothérapie et/ou la radiothérapie. Ceci est notamment démontré dans le cancer du sein justifiant les tentatives de modulation thérapeutique de cette voie dans cette pathologie[21].

2.1.4 Angiogenèse et cellules souches cancéreuses (CSC)

Dans le gliome malin, Bao et collègues ont montré que les tumeurs formées à partir de cellules souches cancéreuses (déterminées sur un marqueur de surface, le CD133 ou prominin) sécrétaient des niveaux plus élevés de facteur de croissance endothélial (VEGF) qui étaient de plus induits par l'hypoxie en comparaison avec les tumeurs issues de cellules qui n'ont pas ce phénotype de cellules souches[22].
Les effets pro-angiogéniques des cellules souches cancéreuses du gliome sont abolis par l'utilisation de l'anticorps neutralisant anti-VEGF, le bevacizumab. Le bevacizumab avait une efficacité anti-angiogénique in vivo et abolissait la croissance des xénogreffes issues de cellules gliales tumorales avec un phénotype de cellules souches alors que son effet était plus limité sur les xénogreffes issues de cellules tumorales qui n'avaient pas ce phénotype. Ces données suggèrent que les cellules tumorales qui ont un phénotype de cellules souches peuvent être un facteur clé de la synthèse de facteurs angiogéniques et que cibler la population de cellules souches avec un traitement anti-angiogénique peut être un facteur clé pour les patients. D'autres auteurs, toujours dans les tumeurs glio-astrocytaires malignes, ont aussi montré qu'en plus d'une radiorésistance les cellules souches cancéreuses stimulaient l'angiogénèse par la sécrétion accrue de VEGF. Cet auteur conclut que les voies de signalisation de l'hypoxie et de maintien des cellules souches sont des cibles idéales pour sensibiliser les cellules souches cancéreuses aux thérapies cytotoxiques et améliorer la prise en charge des patients[23]. Des essais cliniques ont en effet montré une efficacité de l'association bevacizumab à une chimiothérapie de type irinotecan sur les glioblastomes en rechute et les niveaux d'expression de VEGF par les cellules tumorales sont associés à une réponse radiologique, l'hypoxie, la survie mesurée au début du traitement[24-26].

Dans le cancer du sein bien que l'efficacité des traitements anti-angiogéniques ait atteint un niveau de preuve suffisant pour autoriser leur utilisation en situation métastatique, il n'existe pas de données sur cellules souches et angiogénèse. C'est ce que nous nous proposerons de déterminer dans ce projet comme corollaire de l'effet du bévacizumab sur ces mêmes cellules. Les cellules souches déterminées sur le phénotype CD44+/CD24-/Lin- sont résistantes aux chimiothérapies standards données en situation néoadjuvante[27]. Une étude effectuée à partir d'une lignée cellulaire, MCF7, suggère que ces cellules souches seraient aussi résistantes à la radiothérapie[7]. Des nouvelles stratégies thérapeutiques sont donc à développer. Les données des études sur les tumeurs gliales malignes suggèrent que les traitements anti-angiogéniques seraient une perspective prometteuse pour cibler ces cellules souches. De plus dans le cancer du sein inflammatoire qui est un modèle en terme d'angiogénèse et qui sécrète de nombreux facteurs angiogéniques dont le VEGF, des données non encore publiées nous ont permis de mettre en évidence le rôle important de ces cellules souches cancéreuses dans la croissance tumorale mais aussi dans la formation des métastases (Charafe-Jauffret, J Exp Med en révision).

Toutes ces données préliminaires sont en faveur d'un intérêt à l'utilisation du bevacizumab pour cibler ces cellules souches cancéreuses dans le cancer du sein.

2.2 Données des traitements à évaluer

2.2.1 Chimiothérapie néo-adjuvante et cancer du sein

La chimiothérapie néo-adjuvante dite «première» ou encore d'induction correspond à l'utilisation d'un traitement cytotoxique systémique avant le traitement loco-régional. Elle constitue à l'heure actuelle le standard de prise en charge des cancers du sein localement avancé et/ou inflammatoire[28-30] afin d'améliorer le contrôle local et la survie en comparaison à un traitement local exclusif. Elle est également utilisée dans le cancer du sein à tout stade y compris opérable d'emblée[29,31,32] dans l'objectif de permettre un chirurgie conservatrice plus fréquente, de tester la chimiosensibilité tumorale in vivo, d'éradiquer précocement la maladie micro-métastatique. Elle est de plus la situation privilégiée pour intégrer aux essais

cliniques des approches translationnelles portant sur la biologie tumorale, les mécanismes d'action des agents thérapeutiques et la recherche de facteurs prédictifs de la réponse thérapeutique.

Les premières études de chimiothérapie néo-adjuvante ont inclus des protocoles à base d'anthracyclines[33-41] puis dès la fin des années 90 ont incorporé des taxanes soit en association concomitante soit en association séquentielle[42-52].
Plusieurs essais ont comparé des schémas avec ou sans taxanes. L'essai d'Aberdeen[50,51] incluait 162 patientes recevant 4 cycles de CVAP (cyclophosphamide, vincristine, doxorubicine, prednisolone) néo-adjuvant. Les patientes en réponse objective étaient randomisées entre 4 cycles supplémentaires (bras A) ou 4 cycles de docetaxel (bras B) avant chirurgie tandis que les patientes non répondeuses recevaient 4 cycles de docetaxel (bras C). Les taux de réponse objective étaient de 64% dans le bras A, 85% dans le bras B et 47% dans le bras C. Les taux de réponse complète histologique étaient de 15% dans le bras A, 31% dans le bras B et 3% dans le bras C. Les taux de survie sans rechute à 5 ans sont de 72% dans le bras A et 90% dans le bras B tandis que les taux de survie globale à 5 ans sont respectivement de 72% le bras A et 97% dans le bras B. Le taux de conservation mammaire est de 67% dans le bras B versus 48% dans le bras A.

Dans l'essai du NSABP B-27[46,47], 2411 patientes étaient randomisées entres les schémas suivants :
- groupe I : 4 AC suivis de chirurgie et radiothérapie
- groupe II : 4 AC suivis de 4 docetaxel puis chirurgie et radiothérapie
- groupe III : 4 AC suivis de chirurgie puis 4 docetaxel puis radiothérapie

Les taux de réponse objective étaient de 85.7%, 90.5 et 85.4% dans les groupes I, II et III, respectivement tandis que les taux de réponse complète histologique étaient de 12.9, 26 et 14.5% dans les groupes I, II et III respectivement. Les taux de conservation mammaire étaient de 47.9, 50.8 et 48.9% dans les groupes I, II et III respectivement.
Ces deux essais sont en faveur de l'utilisation d'une association séquentielle d'anthracyclines suivi de docetaxel comme stratégie de référence en situation néo-adjuvante.
Un autre essai de Dieras et al[49] a comparé une chimiothérapie d'association doxorubicine-paclitaxel versus une combinaison doxorubicine-cyclophosphamide chez deux cent patientes. Le taux de réponse clinique était de 89% (dont 15% de RC et 16% de réponse complète histologique) dans le bras AP versus 70% (dont 7% de RC et 10% de réponse complète histologique) dans le bras AC. Un traitement conservateur était possible dans 58% des cas dans le bras AP et dans 45% dans le bras AC. Les survies sans rechute étaient comparables. L'essai germanique GEPARDUO[45] impliquant 913 patientes a lui directement comparé une association doxorubicine-docetaxel toutes les 2 semaines 4 cycles à un schéma séquentiel AC 4 cycles suivis de 4 cycles de docetaxel. L'association séquentielle permettait d'obtenir une amélioration du taux de réponse clinique (85 versus 75%), du taux de réponse complète histologique (14% versus 7%) et du taux de chirurgie conservatrice 63 vs 58% (33, 34). Une autre étude[44] a comparé, chez 40 patientes présentant un cancer du sein de stade II et III, une association séquentielle de 3 cycles de doxorubicine 75 mg/m² toutes les 2 semaines suivies de 3 cycles de docetaxel 100 mg/m² toutes les deux semaines à une association doxorubicine 50 mg/m² + docetaxel 75 mg/m² toutes les 3 semaines. Le taux de réponse clinique était similaire dans les deux groupes (87%) avec cependant plus de réponse complète histologique (15.8% versus 9.5%) et plus de conservation mammaire (37% versus 19%) dans le bras séquentiel par rapport au bras concomitant.
Au final pour les cancers opérables traités en situation néo-adjuvante, les taux de réponse objective clinique varient entre 60 et 90% incluant 15 à 60% de réponse complète. La chirurgie conservatrice reste possible dans 50 à 87% des cas. Les taux de réponse complète histologique varient de 7 à 34%. A 5 ans, les taux de survie fluctuent entre 58 et 97%. L'association séquentielle anthracyclines/taxanes semble être le schéma optimal.

Plusieurs essais randomisés ont comparé chimiothérapie néo-adjuvante à une chimiothérapie adjuvante[41,53-59]. Les résultats d'une méta-analyse récente indiquent que la chimiothérapie néo-adjuvante obtient des résultats en terme de survie comparables à la chimiothérapie adjuvante tout en permettant une chirurgie conservatrice plus fréquente[59,60]. De façon récente, le sous-groupe des cancers du sein HER2+ traités en situation néo-adjuvante a pu bénéficier de l'incorporation du trastuzumab aux schémas cytotoxiques conventionnels. Dans ces schémas, il est obtenu de façon assez régulière des taux de réponse complète histologique supérieur ou égal à 50%[60,61]. La possibilité de combiner des schémas à base d'épirubicine et le trastuzumab reste expérimentale même si des résultats spectaculaires ont pu être obtenus avec ce type d'association[62] (NoAH study, SABC meeting 2008).

Ainsi, les stratégies de traitements néo-adjuvants associant de façon séquentielle régime à base d'anthracyclines puis taxanes avec ou sans trastuzumab selon le statut HER2 représentent un tronc commun systémique applicable à la plupart des cancers du sein opérables comme localement avancés.

2.2.2 Bevacizumab et cancer du sein : données précliniques

Le bevacizumab est un anticorps monoclonal humanisé dirigé contre le VEGF composé des régions framework d'une IgG1 humaine et des régions CDR d'un anticorps monoclonal (muMab VEGF A.4.6.1) murin et qui bloque la liaison du VEGF humain à ses récepteurs. Son poids moléculaire est d'environ 149 kDa et il est glycosylé. Il reconnaît tous les isoformes du VEGF avec une affinité d'environ 8.10-10 M mais pas les autres facteurs de croissance peptidiques suivants : fibroblast growth factor, epidermal growth factor, hepatocyte growth factor, nerve growth factor, platelet derived growth factor. Son activité anti-angiogénique est supposée résulter de la liaison au VEGF de son élimination de l'environnement tumoral. Les autres mécanismes qui sous-tendent l'activité anti-tumorale comprennent des modifications qui tendent à la normalisation de la vascularisation tumorale, de la pression interstitielle intratumorale et de la perméabilité vasculaire et qui pourraient favoriser la pénétration de la chimiothérapie intratumorale[21].

Le bevacizumab a montré une synergie en association à la chimiothérapie dans des modèles précliniques de différents types de tumeurs, notamment le cancer du sein. Des études précliniques évaluant l'angiogénèse tumorale par IRM ont montré qu'un anticorps anti-VEGF pouvait inhiber la croissance tumorale et la perméabilité vasculaire tumorale chez des rats athymiques ou nude xenogreffés avec des cellules de cancer du sein humain, la combinaison avec la doxorubicine se révélant plus efficace que chaque agent utilisé seul[63]. Dans un test d'invasion sur matrigel qui utilisait une lignée de cancer du sein humain, le bevacizumab se révélait capable de surmonter l'inhibition de l'activité antiangiogénique du docetaxel induite par le VEGF[64].

En résumé, les principaux mécanismes d'action supposés du bevacizumab sont :
- inhibition de la prolifération des cellules endothéliales au niveau des microcapillaires et régression de la microvascularisation intratumorale[65,66]
- normalisation de la vascularisation intratumorale résiduelle avec réduction de la pression interstitielle et de l'hypoxie intratumorale[65,67].
- blocage des processus de survie cellulaire VEGF-dépendant des cellules endothéliales et potentialisation de l'effet cytotoxique des chimithérapies[65]
- blocage de la mobilisation des progéniteurs endothéliaux circulants et des cellules dérivées de la molle osseuse à activité proangiogénique[65,68]
- effets directs sur les cellules tumorales exprimant VEGFR par blocage des interactions VEGF/VEGFR à l'origine de signaux de survie[65,69].

Cependant, les mécanismes d'action de ce composé restent mal connus et l'hypothèse d'un effet anti-CSC a également été suggérée[22].

2.2.3 Bevacizumab et cancer du sein : données cliniques

2.2.3.1 Bevacizumab et cancer du sein métastatique

Sur la base des données précliniques suggérant une activité du bevacizumab dans les cancers du sein, ce médicament a été d'abord évalué en monothérapie dans une phase I/II d'escalade de dose (AVF0776g) dans des cancers du sein métastatiques. Trois doses (3, 10 et 20 mg/kg toutes les 2 semaines) ont été explorées chez 75 patients en rechute après au moins une première ligne administrée pour la maladie métastatique. Le palier d'inclusion supérieur (20 mg/kg) a été suspendu pour toxicité à type de céphalées, nausées et vomissements chez 25% des patients et un épisode de toxicité cardiaque de grade 3 (insuffisance cardiaque) chez 2 patients avec des antécédents de prétraitement par anthracyclines et d'irradiation de la paroi thoracique gauche. Aucune toxicité mortelle n'a été observée. Les principaux événements indésirables comprenaient HTA, saignements mineurs, quelques événements thrombo-emboliques et protéinurie sans dysfonction rénale. Le taux de réponse objective a été de 6.7% avec 16% de maladie stable, une durée médiane de réponse de 5.6 mois et une survie globale de 10 mois. La dose de 10 mg/kg toutes les 2 semaines a été retenue comme celle présentant le meilleur ratio bénéfice risque avec une tolérance correcte et sur le plan anti-tumoral 1 réponse complète et 2 réponses partielles.[70]
Un premier essai randomisé a comparé chez 462 patientes (AVF2119g) présentant un cancer du sein métastatique déjà prétraitées par anthracyclines et taxanes, capecitabine orale 2500 mg/m² 14 jours/21 jours versus une association capecitabine orale plus bevacizumab 15 mg/3semaines. Cet essai a démontré une augmentation significative du taux de réponse (19.8% vs 9.1%, p =0.001) sans différence significative en terme de survie sans progression ou de survie globale[52]. La tolérance était correcte sans aggravation des toxicités habituelles de la capecitabine[71].

Un second essai randomisé (E2100) a comparé, chez 722 patientes présentant un cancer du sein métastatique HER2- en première ligne thérapeutique, un traitement par paclitaxel hebdomadaire 90 mg/m² J1, J8, J15 (J1=J28) et une association paclitaxel bevacizumab 10 mg/kg tous les 15 jours. Le taux de réponse était doublé dans le bras bevacizumab (de 25.2 à 49.2%, p < 0.001) comme l'était la médiane de survie sans progression (5.9 vs 11.8 mois, HR=0.6, p< 0.001). Cependant, les survies globales n'apparaissaient pas significativement différentes (25.2 vs 26.7 mois, HR=0.88, p=0.16). HTA, protéinurie, céphalées, AVC ischémique, neuropathie périphérique (lié à un temps de traitement par chimiothérapie plus long) étaient plus fréquents dans le bras bevacizumab, ainsi que les épisodes infectieux, même si les neutropénies étaient rares dans les deux bras.[72]

Un dernier essai randomisé récemment présenté à l'ASCO 2008 évaluait chez 736 patientes présentant un cancer du sein métastatique HER2- en première ligne thérapeutique un traitement par docetaxel 100 mg/m² + bevacizumab 7.5 ou 15 mg/kg tous les 21 jours versus docetaxel seul. Le taux de réponse était de 44% pour le docetaxel seul versus 55 (p=0.029) et 63% (p=0.0001) pour des doses croissantes de bevacizumab ; la survie sans progression était significativement améliorée dans chacun des bras d'association passant de 8 à 8.7 (p=0.03) ou 8.8 mois (p=0.0099). Les données de survie globale ne sont pas suffisamment matures. La tolérance restait correcte dans le bras combiné avec cependant un excès modeste de neutropénie et d'infection, d'HTA (bras 15 mg/kg) et de neuropathie.[73]

De nombreux essais combinant bevacizumab et chimiothérapie ou autres thérapies ciblées sont en cours dans les cancers du sein métastatiques.

2.2.3.2 Bevacizumab et cancer du sein non métastatique : association aux anthracyclines

Un essai à deux bras non randomisé, destiné à évaluer les faisabilités de l'incorporation du bevacizumab aux protocoles adjuvants à base d'anthracyclines, a été présenté à San

Antonio en 2007 et concernait 226 patientes présentant un cancer du sein avec envahissement ganglionnaire axillaire (Miller et al, San Antonio Breast cancer meeting, 2007). Les patientes recevaient un schéma doxorubicine-cyclophosphamide dose dense suivi de paclitaxel en association au bevacizumab 10 mg/kg tous les 15 jours x 26 démarré avec AC (bras A, ddBAC>BT>B, n=104) ou avec paclitaxel (Bras B, ddAC>BT>B; n=122). Chaque fois qu'indiqué, radiothérapie et traitement hormonal étaient administrés de façon concomitante au bevacizumab. Le critère de jugement principal était l'incidence des dysfonctions cardiaques. Avec un suivi court de 9.1 mois pour le bras A et 5.3 mois pour le bras B, 4 événements d'insuffisance cardiaque ont été rapportés après 4 (n=2), 6 (n=1) et 17 cycles (n=1) auxquels s'ajoute un cas d'insuffisance cardiaque à 3 mois de la fin du traitement chez un patient ayant interrompu le traitement après 9 cycles. Une fraction d'éjection ventriculaire gauche (FEVG) < 50% a été documentée chez 3 des 5 patients. La FEVG médiane pré-thérapeutique était de 65% dans les 2 bras et était de 61.5 et 62.5% dans les bras A et B après 8 cycles. Une myélosuppression était fréquemment retrouvée (25% de neutropénie G3/4) mais les neutropénies fébriles étaient rares. HTA et thromboses de G3 ou plus étaient rares (1%) et aucune protéinurie G3 ou plus n'a été observée. Les auteurs ont conclu à la faisabilité de cette association avec la nécessaire poursuite de la surveillance cardiaque.

Wedam et al.[74] ont réalisé une étude concernant 21 patientes présentant un cancer du sein inflammatoire ou localement avancé recevant un traitement néo-adjuvant et adjuvant à base de bevacizumab. Les patientes recevaient bevacizumab 15 mg/kg à J1 puis 3 semaines plus tard débutait une association bevacizumab/doxorubicin 50 mg/m²/docetaxel 75 mg/m² toutes les 3 semaines 6 cycles précédant la chirurgie. Au décours, elles recevaient 8 cycles de bevacizumab et une hormonothérapie. Une HTA G3 nécessitant une intervention thérapeutique a été notée chez 8 patients sur 21 tandis que 3 patients avec une HTA à l'entrée ont nécessité une majoration thérapeutique. Des troubles de la cicatrisation post-opératoire ont été observés chez 5 patients. Le taux de réponse clinique était de 67% avec cependant aucune réponse complète. Les données pharmacodynamiques présentées[75], qu'il s'agisse des modifications de la perméabilité vasculaire en IRM-DCE, du niveau de phosphorylation du VEGFR2 ou encore du niveau d'induction de l'apoptose, attestaient de la réalité d'un effet anti-angiogénique.

De nombreux essais combinant chimiothérapie et bevacizumab sont à l'heure actuelle en cours de réalisation en situation adjuvante et/ou néoadjuvante[76-78]

2.3 Justification de l'essai

L'un des postulats essentiels de l'hypothèse des cellules souches cancéreuses (CSC) est leur contribution critique au phénomène de chimiorésistance et éventuellement à la rechute. De nombreuses études in vitro ont montré que ce contingent cellulaire présentait une relative quiescence et exprimait fortement des molécules d'efflux des xénobiotiques pouvant expliquer la capacité à résister aux cytotoxiques conventionnels[79-81].
Très récemment, une étude clinique originale a exploré l'évolution du contingent CSC au sein de tumeurs provenant de patientes bénéficiant d'un traitement néo-adjuvant[27]. De manière très intéressante sous traitement cytotoxique conventionnel, un enrichissement en CSC identifiées par marquage CD44+/CD24- a été observé au niveau du matériel tumoral analysé, confirmé par leur plus grande capacité à reconstituer une authentique tumeur dans un modèle de xénogreffe murine. Ceci suggérait que ce contingent était effectivement chimio-résistant et pouvait donc faire le lit d'une récidive tumorale. De façon importante, des patientes présentant une tumeur HER2+ et recevant un traitement par lapatinib, un inhibiteur de tyrosine kinase anti-HER2 et anti-EGFR, ont été soumises aux mêmes analyses : dans ces cas, il n'était pas détecté d'augmentation significative du pourcentage de cellules CD44+/CD24- et même une tendance à la baisse de ce pourcentage, quoique non significative, était observée. Ces résultats démontrent qu'il est donc possible de monitorer le

contingent CSC in vivo par des techniques de marquage simple, qu'il est encore possible d'optimiser et que ce contingent semble être enrichi par les stratégies de traitements cytotoxiques conventionnelles tandis que l'utilisation d'une thérapie ciblant des acteurs moléculaires dont le rôle peut être critique dans la biologie des cellules souches pourrait traiter efficacement ce contingent.

Comme rappelé dans le paragraphe précédent, une collaboration développée entre notre Institut et le laboratoire du Pr Max Wicha, (Ann Arbor, Michigan, USA) qui fait référence dans le domaine des CSC a permis de montrer que le marquage IHC de l'ALH1 constitue un marqueur fiable de contingent CSC[82]. Notre hypothèse est donc que le suivi de l'évolution de ce marquage sous traitement peut constituer un «surrogate endpoint» permettant de documenter l'activité anti-cellules souches des composés thérapeutiques à évaluer.

Des données récentes suggèrent une interaction complexe entre CSC et angiogénèse, les CSC sécrétant du VEGF et des facteurs pro-angiogéniques tandis que les CSC pourraient résider dans des niches vasculaires VEGF-dépendantes[83,84]. Notre hypothèse est donc que le bevacizumab, un anticorps anti-VEGF humanisé récemment enregistré en combinaison à la chimiothérapie dans le traitement des cancers du sein métastatiques et en cours d'évaluation en situation adjuvante et néo-adjuvante, pourrait permettre, en association à la chimiothérapie conventionnelle, de cibler le contingent CSC non affecté par les cytotoxiques. Il s'agira donc d'un essai preuve du concept pouvant rentrer dans la catégorie des essais de phase II permettant de valider ou non l'activité biologique anti-CSC de ce composé.

Rationnel pour le schéma expérimental
- La stratégie néo-adjuvante par une association séquentielle FEC/docetaxel peut être considérée comme un standard thérapeutique dans les cancers du sein localement avancés ou inflammatoires et comme une option tout à fait validée dans les cancers du sein opérables d'emblée.

- Un agent anti-angiogénique tel que le bevacizumab pourrait avoir une activité anti-CSC et donc s'opposer à l'enrichissement en CSC induit par les cytotoxiques conventionnels.

- Des données récentes montrent que la combinaison FEC/bevacizumab apparaît faisable en situation adjuvante et néo-adjuvante. La dose de bevacizumab utilisée correspond à celle évaluée dans ces études.

- La randomisation avec un groupe contrôle est nécessaire pour valider dans le bras cytotoxique seul et par marquage ALDH1 l'enrichissement en CSC suggéré par l'étude de Li et al.

2.4 Perspectives

Ce projet permet de valider la mise au point d'une plateforme de monitorage pharmacodynamique des CSC comprenant :
- évaluation en IHC sur tissus fixés du marquage ALDH1et CD44/CD24
- évaluation en cytométrie de flux sur tissu frais dissocié de l'activité ALDEFLUOR et du pourcentage de cellules CD44/CD24/lin
- évaluation de la sécrétion par les cellules souches cancéreuses du facteur pro-angiogénique VEGF - comparaison avec la population tumorale plus différenciée - monitoring de ce statut sous traitement si possible (co-marquage ALDEFLUOR et VEGF).

Par ailleurs, des études ancillaires utilisant notamment les outils d'analyse du transcriptome et du protéome seront associées à ce projet dans le but d'identifier de façon exploratoire des signatures moléculaires permettant de prédire un bénéfice au bevacizumab et/ou l'activité anti-CSC.

Si la preuve du concept est faite, cet essai pourrait permettre d'envisager l'utilisation de cette plateforme pour rechercher et valider in vivo d'autres approches thérapeutiques permettant de cibler efficacement le contingent de CSC.

3. OBJECTIFS DE LA RECHERCHE

3.1 Objectif principal

- Evaluer l'activité anti-Cellules Souches Cancéreuses (CSC) après traitement par bevacizumab en combinaison à la chimiothérapie cytotoxique dans les tumeurs du sein candidates à un traitement néo-adjuvant.

3.2 Objectifs secondaires

- Evaluer la tolérance de l'association chimiothérapie + bevacizumab
- Evaluer le taux de réponse complète histologique de l'association chimiothérapie + bevacizumab
- Estimer la survie sans maladie, sans rechute et la survie globale
- Etudes ancillaires :
  - Corrélation entre marquage ALDH1 et CD44/CD24 en IHC
  - Nombre de cellules CTC ~~et CEC~~
  - Transcriptomique
  - Protéomique
  - Polymorphisme du VEGF
  - Imagerie

4. CONCEPTION DE LA RECHERCHE

4.1 Critère d'évaluation

L'activité anti-Cellules Souches Cancéreuses (CSC) est mesurée par le pourcentage de cellules ALDH1 + résiduelles après 4 cycles de traitement.

4.2 Méthodologie et présentation schématique

Essai de phase II randomisé, ouvert, oligocentrique, national.

Les patients inclus dans l'essai auront du fait du protocole l'ajout ou non de bévacizumab pendant les 8 cycles de chimiothérapie standard, ainsi que la réalisation d'une macrobiopsie après 4 cycles de traitement.
Un prélèvement sanguin sera réalisé pour les personnes qui y consentent avant traitement, après le premier cycle et après les 8 cycles de chimiothérapie. La réalisation d'une échographie mammaire avec injection de produit de contraste pourra aussi être proposée.

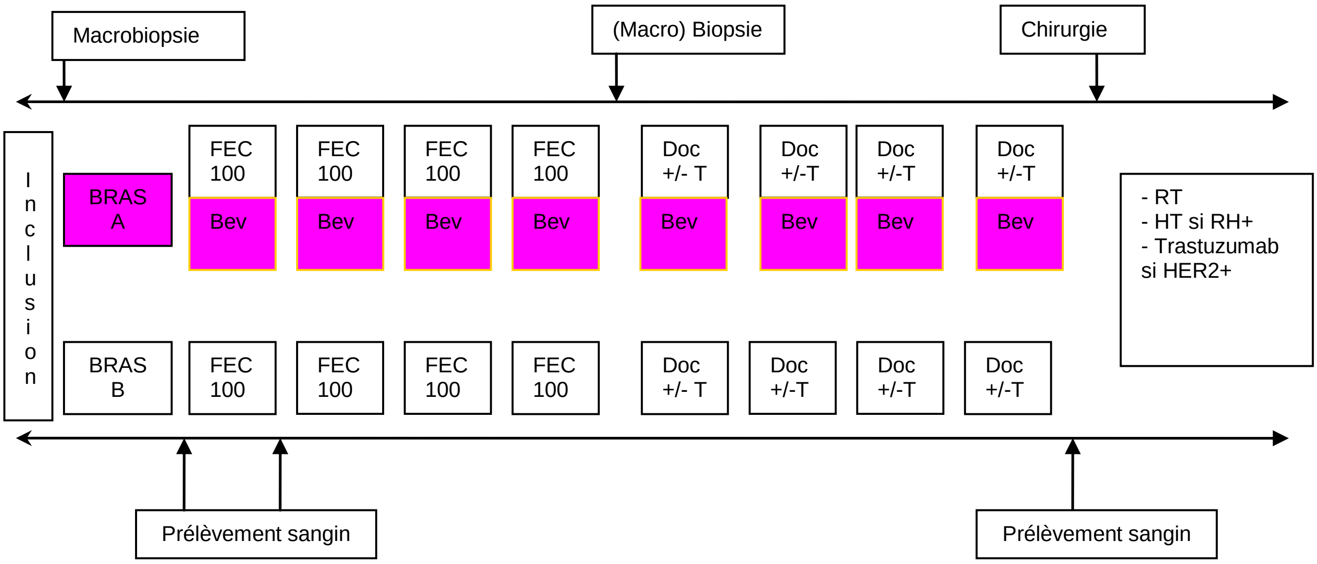

Doc : docetaxel, T : Trastuzumab, Bev : bevacizumab ; RT : radiothérapie ; HT : hormonothérapie

4.3 Mesures prises pour éviter ou réduire les biais

L'attribution du bras de traitement sera déterminée après l'inclusion par randomisation à partir d'un tirage aléatoire soumis aux contraintes suivantes :
- la ratio de bras B attribué par la randomisation sera de 1:2
- le tirage sera stratifié sur le statut HER2 à l'inclusion
- la liste de randomisation sera générée par blocs de taille aléatoire de sorte que l'équilibre recherché soit garanti en cas d'arrêt prématuré de l'étude.

4.4 Posologie, modalités d'administration, conditionnement, étiquetage

Les traitements seront administrés en unité de jour ou en hospitalisation conventionnelle.

Deux bras de traitement seront étudiés :

Bras A : bras expérimental

- 4 cycles de FEC100/**bevacizumab** administrés tous les 21 jours :
  5FU 500 mg/m² IV en 30 minutes,
  Epirubicine 100 mg/m² IV en 15 minutes,
  Cyclophosphamide 500 mg/m² IV en 30 minutes
  Bevacizumab 15 mg/kg en perfusion IV

- puis 4 cycles de docetaxel/ **bevacizumab** tous les 21 jours :
  Docetaxel 100mg/m² IV en 1 heure
  Bevacizumab 15 mg/kg en perfusion IV.

Les tumeurs surexprimant HER2 (IHC3+ et/ou FISH/CISH+) recevront en association au docetaxel/ bevacizumab un traitement par trastuzumab.

Bras B : traitement de référence

- 4 cycles de FEC100 administrés tous les 21 jours :
  5FU 500 mg/m² IV,
  Epirubicine 100 mg/m² IV,
  Cyclophosphamide 500 mg/m² IV
- puis 4 cycles de docetaxel 100mg/m² IV en 1 heure tous les 21 jours.

Les tumeurs surexprimant HER2 (IHC3+ et/ou FISH/CISH+) recevront en association au docetaxel un traitement par trastuzumab.

Le bevacizumab et le trastuzumab pourront être administrés avant ou après la chimiothérapie.

Les traitements cytotoxiques seront réalisés si les polynucléaires neutrophiles ≥ 1 000/mm^3 et les plaquettes ≥ 100 000/mm^3 ; si ces conditions ne sont pas requises, le traitement pourra être reporté de 21 jours maximum.

Adaptation des doses
Les adaptations protocolaires concernent uniquement le bevacizumab (cf annexe 5) ; pour les autres produits, le traitement sera conduit à discrétion de l'investigateur.

En cas de retard à l'administration de la chimiothérapie, la perfusion de bevacizumab sera décalée à la date de réalisation de la chimiothérapie.

En cas de retard à l'administration du bevacizumab, la chimiothérapie sera administrée sans délai et le bevacizumab sera décalé au cycle de chimiothérapie suivant.

Aucune réduction de posologie du bevacizumab ne sera réalisée. La gestion des toxicités spécifiques du bevacizumab sera réalisée comme décrit en annexe 5.

4.5 Durée de participation des personnes et chronologie des périodes de l'essai

Etape 1 :
- Réalisation d'une biopsie initiale (macrobiopsie si possible) pour congélation et fixation,
- Début du traitement comprenant 4 cycles à 3 semaines d'intervalle associant :

Bras A (FEC100 + bevacizumab) ou **Bras B** (FEC100 seul)
- Après le 4ème cycle (dans la 12ème semaine de traitement), réalisation d'une nouvelle biopsie tumorale.

Etape 2 :
- Début à 3 semaines du dernier cycle de FEC 100, des 4 cycles à 3 semaines d'intervalle comportant :

Bras A (docetaxel + bevacizumab) ou **Bras B** (docetaxel seul)
- en association avec le trastuzumab si la tumeur surexprime HER2 à 8 mg/kg (1ère administration) puis 6 mg/kg (administrations suivantes).

Etape 3 : **La chirurgie** devra être réalisée entre 3 à 6 semaines après la dernière cure de chimiothérapie (J169-J190).

Suivi et prise en charge après la chirurgie
La prise en charge des patientes sera ensuite déterminée à la discrétion de l'investigateur et comprendra radiothérapie, poursuite éventuelle du trastuzumab, introduction de l'hormonothérapie si indiqué. Le suivi des personnes sera réalisé pendant 5 ans.
Les patientes présentant un cancer du sein métastatique synchrone randomisées dans le bras sans bevacizumab pourront recevoir du bevacizumab après la chirurgie ; une surveillance particulière sera réalisée à la discrétion de l'investigateur.

4.6 Description des règles d'arrêt du traitement

Les patients peuvent arrêter prématurément le traitement de l'étude (chimiothérapie +/- bevacizumab) pour les raisons suivantes :

- toxicité,
- progression de la maladie,
- retrait de consentement,
- perdu de vue,
- violation majeure de protocole.

En dehors d'un retrait de consentement, un arrêt prématuré du traitement n'entraîne pas un arrêt de l'étude. Dans la mesure du possible, les patients ayant arrêté prématurément leur traitement seront suivis selon les mêmes modalités que les autres patients, en particulier le bilan d'évaluation post traitement sera réalisé.

5. SELECTION DES PERSONNES DE LA RECHERCHE

5.1 Critères d'inclusion

1 - Cancer du sein histologiquement confirmé, de grade > I, de taille > 3 cm, quelque soit le statut HER2 et la réceptivité hormonale, avec tumeur primaire en place opérable ou non opérable d'emblée et candidat à une chimiothérapie néo-adjuvante.
Les cancers du sein métastatiques synchrones et inflammatoires sont éligibles.
2 - Age de 18 ans ou plus
3 - Performance Status 0 à 1
4 - Espérance de vie ≥ 3 mois
5 - Capable de compliance au protocole
6 - Patient affilié à un régime de sécurité sociale ou bénéficiaire d'un tel régime
7 - Consentement éclairé signé
8 - Fonctions organiques satisfaisantes :

- Fonction hématologique satisfaisante:
  Nombre absolu de neutrophiles ≥ 1,2 x 10^9/L ET
  Plaquettes ≥ 100 x 10^9/L ET
  Hb ≥ 9 g/dl
- Fonction hépatique satisfaisante :
  Bilirubine totale ≤ 1,5 LSN sauf si l'élévation est due à une maladie de Gilbert ou à un syndrome similaire entraînant une conjugaison lente de la bilirubine ET
  ASAT < 2,5 LSN ET ALAT < 2,5 LSN chez les patients sans métastases hépatiques ; <5 LSN chez les patients avec métastases hépatiques
- Fonction rénale satisfaisante :
  Créatininémie ≤ 1.25 LSN ou clairance de la créatinine ≥ 50 ml/min selon la formule de Cockroft et Gault ET
  Bandelette urinaire pour la protéinurie < 2+. Si, à l'inclusion, la protéinurie est ≥ 2+ à la bandelette, un recueil des urines sur 24 heures sera réalisé et devra montrer une protéinurie ≤ 1 g en 24 heures.
- Coagulation normale :
  Chez les patients ne recevant pas d'anticoagulants :
  INR (International normalized ratio) ≤ 1,5 (sauf pour les patientes traitées par un anticoagulant à dose préventive) ET
  TCA ≤ 1,5 x LSN au cours des 7 jours précédant l'inclusion

Note : Les patients recevant des anticoagulants à doses curatives peuvent être inclus à condition que le niveau d'anticoagulation soit stable depuis au minimum 2 semaines avant l'entrée dans l'étude et que les tests de coagulation se trouvent dans les normes thérapeutiques.

- Fonction cardiaque satisfaisante : Fraction d'éjection ventriculaire gauche (FEVG) ≥ 55% (mesure isotopique ou échographique).

5.2 Critères de non-inclusion

1 - Cancer du sein lobulaire (sauf si ils sont de grade 3, ou si ils surexpriment HER2, ou si ils sont inflammatoires ou métastatiques), de grade I (sauf si ils surexpriment HER2, ou si ils sont inflammatoires ou métastatiques) ou multifocal.
2 - Antécédents de cancer (autres que les cancers cutanées de type basal et épidermoïdes ou cancer in situ du col utérin traités de façon curative) dans les 5 années précédent l'entrée dans l'étude
3 - Antécédent de traitement par bevacizumab
4 - Femme enceinte, allaitante, ou non ménopausée avec utérus intact n'utilisant pas de moyens de contraception efficace
5 - Patient socialement ou psychologiquement instable, incapable de compliance au traitement prévu ou à la surveillance requise pour des raisons géographiques, sociales ou psychiques
6 - Antécédents de diathèse hémorragique ou de coagulopathie héréditaire
7 - HTA non contrôlée (systolique > 150 mmHg et/ou diastolique > 100 mmHg) avec ou sans traitement. Les patients avec une HTA initiale sont éligibles dès normalisation des chiffres sous traitement
8 - Affection cardiovasculaire évolutive telle que : Infarctus du myocarde dans les 6 derniers mois, angor instable, insuffisance cardiaque congestive NYHA ≥ II, arythmie cardiaque sévère non contrôlée ou requérant un traitement spécifique pouvant interférer avec les traitements à l'étude
9 - Antécédents d'événements thrombotiques dans les 6 mois précédent l'entrée dans l'étude (ex : AVC, AIT)
10 - Dyspnée au repos secondaire à une complication lié au cancer ou toute autre pathologie qui génère une oxygéno-dépendance
11 - Patiente diabétique traitée par des antidiabétiques oraux ou par l'insuline, avec une cardiopathie sous-jacente à l'échographie.
12- Infection évolutive (nécessitant une antibiothérapie par voie IV) ou toute autre affection neurologique, métabolique, ou toute anomalie clinique ou biologique faisant redouter un état contre-indiquant l'utilisation de l'un des médicaments à l'étude ou qui pourrait augmenter le risque de complications liées au traitement.
13 - Antécédents chirurgicaux : chirurgie ou traumatisme majeur dans les 28 jours précédent l'entrée dans l'étude, chirurgie mineure dans les 24 heures précédent la première injection de bevacizumab
14 - Plaie, ulcère actif ou fracture osseuse non cicatrisés
15 - Antécédent de fistule abdominale, trachéo-œsophagienne, ou tout autre fistule non digestive, antécédent de perforation digestive ou d'abcès intra-abdominal dans les 6 mois précédent l'inclusion
16 - Co-médications : utilisation récente ou au long cours de tout AINS (aspirine > 325 mg/jour) ou de tout antiagrégant plaquettaire (dipyridamole, ticlopidine, clopidogrel > 75 mg/jour), ou de tout thrombolytique dans les 10 jours précédent la première administration de bevacizumab.
17 - Traitement expérimental dans les 28 jours précédents l'inclusion
18 - Allergies : allergie connue aux anticorps monoclonaux (bevacizumab, trastuzumab), aux protéines murines, aux produits dérivés de cellules CHO ou aux agents de chimiothérapies de l'étude ou à l'un de leurs excipients.
19 - Patient déjà inclus dans un autre essai thérapeutique avec une molécule expérimentale,
20 - Personnes privées de liberté ou sous tutelle (y compris la curatelle).

5.3 Enregistrement des patients

Après signature du formulaire de consentement et validation des résultats du bilan initial d'inclusion, les patients éligibles sont enregistrés en contactant le promoteur qui assure la centralisation des inclusions :

Bureau d'Etudes Cliniques
Téléphone : 04.91.22.37.78
Fax : 04.91.22.36.01
e-mail : bec@marseille.fnclcc.fr

Un numéro d'inclusion est retourné à l'investigateur.
Le bras de traitement est ensuite attribué par randomisation dès que le statut HER est connu, le traitement doit débuter dans les 15 jours suivant la randomisation dans l'essai.

6. TRAITEMENTS

6.1 Description des traitements nécessaires à la recherche

Traitements expérimentaux

DCI +/- nom	Formulation	Voie	Posologie
Bevacizumab/ Avastin®	Solution	IV	15 mg/kg en 90 minutes, 60 puis 30 minutes, en fonction de la tolérance, toutes les 3 semaines
Epirubicine	Poudre pour solution IV	IV	100 mg/m² en 15 minutes toutes les 3 semaines
Cyclophos-phamide	Poudre pour solution IV	IV	500 mg/m² en 30 minutes toutes les 3 semaines
5FU	Solution	IV	500 mg/m² en 30 minutes toutes les 3 semaines
Docetaxel	Solution	IV	100 mg/m² en 1 heure toutes les 3 semaines

Traitements standards

DCI	Formulation	Voie	Posologie
Trastuzumab/ Herceptin®	Poudre pour solution IV	IV	8 mg/kg en 90 minutes puis 6 mg/kg, en fonction de la tolérance, en 30 minutes toutes les 3 semaines

6.2 Traitements autorisés et interdits

Aucune autre chimiothérapie cytotoxique, aucun inhibiteur des voies de signalisation de Ras/Raf, MEK, AKT kinase et mTOR et aucun traitement immunomodulateur à visée antitumorale n'est permis pendant cette étude.

Pas de vaccination par vaccins vivants, ni de vaccination contre fièvre jaune durant la période de traitement.

Pas d'administration de phenytoïne (Di-hydan®, Dilantin®) durant la période de traitement.

Aucune radiothérapie ou hormonothérapie autre que celle prévue dans l'étude n'est permise.
Toute thérapeutique à visée potentiellement antiangiogénique est interdite.
Les corticoïdes, les biphosphonates sont autorisés.

En cas d'événements thrombo-emboliques veineux, les traitements anticoagulants curatifs sont autorisés, et le traitement par bevacizumab sera suspendu.
L'utilisation d'une anticoagulation prophylactique est autorisée.

6.3 Conditions de stockage, distribution

Le pharmacien de l'établissement de soins sera responsable de la réception, du stockage, de la dispensation et de la gestion des produits soumis à l'étude.
Les médicaments sont dispensés par la pharmacie de manière nominative sous la responsabilité du pharmacien.
Une traçabilité de tous les produits utilisés et fournis par le promoteur dans le cadre des essais cliniques sera assurée pendant toute la durée de l'étude.
La distribution du bevacizumab aux différentes pharmacies des établissements de santé sera réalisée sous la responsabilité du promoteur conformément aux Bonnes Pratiques de Distribution.
Le pharmacien de l'établissement de soins accuse réception de tous les envois en retournant au distributeur un accusé de réception dûment complété.
Le bevacizumab doit être stocké dans un local fermé à clés et à accès limité, à une température n'excédant pas 25°C. Le pharmacien de l'établissement établira une comptabilité des flacons délivrés, utilisés, inutilisés et/ou retournés.

Le bevacizumab sera fourni par le promoteur et sera étiqueté conformément à l'annexe 13 du guide communautaire des Bonnes Pratiques de Fabrication (révisée et adoptée en juillet 2003 par la Commission Européenne). Les autres produits conventionnels seront fournis par les pharmacies des établissements de santé participant à l'essai.

6.4 Procédure de comptabilité

L'attaché de recherche clinique mandaté par le promoteur vérifiera la comptabilité du médicament fourni par le promoteur (Bevacizumab) et s'assurera qu'un formulaire de comptabilité a été validé et signé par le pharmacien de l'établissement.
Les conditionnements seront détruits après utilisation et les certificats de destruction devront être conservés dans le dossier pharmacie du centre investigateur.

7. CRITERES D'EVALUATION

7.1 Description des paramètres

7.1.1 Activité anti-cellules souches cancéreuses

Le critère principal de jugement est la mesure du pourcentage de cellules ALDH1+ en immunohistochimie avant et après 4 cycles de traitement dans chacun des groupes avec et sans bevacizumab. Le marquage sera réalisé comme décrit en annexe 3.

7.1.2 Tolérance

Pour être jugés évaluables pour la toxicité, les patients doivent avoir reçu au moins une injection de FEC100 +/- bevacizumab. Tous les événements indésirables seront recueillis et seront analysés en termes de fréquence et d'intensité. Les toxicités seront évaluées à chaque cycle soit toutes les 3 semaines pendant la phase de chimiothérapie et jusqu'à un mois après la chirurgie. De plus, la tolérance cardiaque sera évaluée à long terme par un ECG et une échographie cardiaque réalisés 12 mois après le début du traitement.

La toxicité est évaluée selon l'échelle de toxicité CTC-AE V3.0 (annexe 6). La tolérance cardiaque sera appréciée selon la classification de la NYHA (annexe 7).

7.1.3 Taux de réponse complète histologique

Le pourcentage de patients présentant une réponse complète histologique sera déterminé. La réponse histologique sera définie sur la pièce opératoire selon la classification de Sataloff*.

Effet thérapeutique basé sur :

Modifications microscopiques avec présence de :
- fibroplasie,
- nécrose,
- modifications myxoides,
- hémorragies,
- dépôt d'hémosidérine,
- foyers de calcification,
- macrophages « mousseux » avec ou sans infiltrat inflammatoire polymorphe.

Score de A à D :

Lésion principale :

T-A : Effet thérapeutique complet ou presque complet
T-B : Subjectivement supérieur à 50% mais inférieur à un effet thérapeutique complet ou presque complet
T-C : Effet thérapeutique inférieur à 50% mais effet évident
T-D : Pas d'effet thérapeutique

Ganglion lymphatique :

N-A : Effet thérapeutique évident, absence de maladie métastatique
N-B : Absence de métastase ganglionnaire ou d'effet thérapeutique
N-C : Présence d'un effet thérapeutique mais présence de métastases ganglionnaires
N-D : Maladie métastatique viable, absence d'effet thérapeutique.

**Sataloff DM, Mason BA, Prestipino AJ, Seinige UL, Lieber CP, Baloch Z. Pathologic response to induction chemotherapy in locally advanced carcinoma of the breast: a determinant of outcome. J Am Coll Surg. 1995 Mar;180(3):297-30.*

7.1.4 Taux de survie sans maladie, survie sans rechute et survie globale

Les survies sans maladie, sans rechute et globale seront estimées à 3 et 5 ans après l'inclusion.

La survie sans maladie est définie comme le temps entre le début du traitement et la rechute locale, contro-latérale, régionale ou métastatique, ou un second cancer primitif (excepté un cancer cutanée épidermoïde ou basal, un cancer in situ du col utérin ou du colon ou un cancer lobulaire in situ du sein) ou le décès quelque soit la cause.

La survie sans rechute est définie comme le temps entre le début du traitement et la rechute locale, régionale ou à distance (incluant tissus mou et ganglions lymphatiques).

La survie globale est définie comme le temps entre le début du traitement et le décès du patient quelle qu'en soit la cause.

7.1.5 Analyses ancillaires

Les principes des approches ancillaires prévues sont décrits en annexe 4 :
- Corrélation entre marquage ALDH1 et CD44/CD24 en IHC
- Nombre de cellules CTC par système CELLSEARCH® avant et après traitement
- ~~Nombre de cellules CEC~~
- Transcriptomique avant et après traitement

- Protéomique plasmatique et tissulaire avant et après traitement
- Détermination du polymorphisme du VEGF sur cellules mononucléées
- Evaluation des modifications de l'angiogénèse tumorale mammaire par échographie à produit de contraste

7.2 Méthodes et calendrier prévus

La surveillance et le calendrier des évaluations sont décrits en annexe 1.

7.2.1 Bilan d'inclusion

Les patients éligibles pour l'essai et ayant signé leur consentement de participation devront subir un bilan initial dans le mois précédant le début du traitement.
Une mention spécifique pour les études ancillaires est prévue dans le consentement.

- **Examen clinique**

Examen clinique avec détermination du poids, de la taille et de la surface corporelle, histoire de la maladie et antécédents, traitements concomitants
Indice de Performance Status (annexe 2)
Signes vitaux (pouls, tension).

- **Examens para-cliniques**

Echo-mammographie (dans les 2 mois précédent le début du traitement)
Scanner thoracique, abdominal et pelvien (ou IRM ou TEP-TDM)
Scintigraphie osseuse (sauf si TEP-TDM)
Scanner cérébral si point d'appel clinique
ECG, Echographie cardiaque
Echographie mammaire avec injection de produit de contraste (étude ancillaire)

- **Biopsie tumorale**

- Réalisation d'une macrobiopsie sous mammotome avec congélation en azote liquide d'une partie du matériel pour les analyses translationnelles et fixation des autres fragments avant inclusion en paraffine (annexe 3).
A l'Institut Paoli-Calmettes, du tissu frais sera obtenu et soumis à une procédure de dissociation pour éventuelle xénogreffe selon le mode opératoire décrit en annexe 4.
- Typage HER2, RE, RP selon les procédures standards.

- **Examens biologiques**

Hématologie : NFP, TP, TCA, INR
Ionogramme sanguin, calcémie, LDH, phosphates, albuminémie, protidémie,
Bilan hépatique (bilirubine, ALAT, ASAT, PAL, GGT),
Bilan rénal (créatinine, urée), bandelette urinaire et recherche de protéinurie des 24 heures si > 2+
Marqueurs tumoraux (ACE, CA 15-3)

- **Prélèvements sanguins en vue des analyses translationnelles**

- ~~2 tubes~~ 1 tube de 10 ml de sang total pour traitement immédiat (recherche de CTC ~~et CEC~~ système Veridex®, CellSearch),

- 2 tubes EDTA et 2 tubes secs de 5 ml seront prélevés et techniqués avant congélation : 5 ml de sérum et 5 ml de plasma plus culot cellulaire sec avec congélation immédiate seront conservés dans les centres à -80°C ou en azote liquide jusqu'à l'analyse finale (protéomique et polymorphisme VEGF)

7.2.2 Bilan de suivi pendant la phase de chimiothérapie

- **Avant chaque cycle**

- Signes vitaux (pouls, TA), examen clinique
- Bandelette urinaire (pour les 8 cycles comportant du bevacizumab)
- Hématologie : NFP, TP, TCA, INR
- Ionogramme sanguin, calcémie, phosphates, albuminémie, protidémie, LDH,
- Bilan hépatique (bilirubine, ALAT, ASAT, PAL, GGT)
- Bilan rénal (créatinine, urée)
- Poids avant le cycle 5

- **Echographie cardiaque, ECG** avant l'injection de bevacizumab du cycle 5 et avant la chirurgie, ainsi que 12 mois après le début du traitement.

- **Biopsie tumorale** (si possible macrobiopsie) après les 4 cycles de FEC100/bevacizumab ou FEC100 (et avant le premier cycle de docetaxel (J78 et J85)).

Congélation en azote liquide d'une partie du matériel pour les analyses translationnelles et fixation des autres fragments avant inclusion en paraffine (annexes 3 et 4).
A l'Institut Paoli-Calmettes, du tissu frais sera obtenu si possible et soumis à une procédure de dissociation selon le mode opératoire décrit en annexe 4.

- **Prélèvements sanguins en vue des analyses translationnelles**

Les prélèvements sont les mêmes que pour le bilan initial, et sont réalisés :
- Avant le deuxième cycle de FEC100/bevacizumab ou FEC100 (à J22)
- Et 14 à 21 jours après le dernier cycle de docetaxel (entre J162 et J169)

En cas de décalage des cycles de chimiothérapie par rapport aux jours théoriques, les prélèvements seront réalisés par rapport aux cycles plutôt que par rapport aux dates.

- **Echographie mammaire avec injection de produit de contraste (étude ancillaire)**

- Avant le deuxième cycle de FEC100/bevacizumab ou FEC100 (à J22),
- Avant le premier cycle de docetaxel (J85)
- Et 14 à 21 jours après le dernier cycle de docetaxel (entre J162 et J169).

- **Evénements indésirables**

Tous les examens révélant une toxicité liée aux traitements de l'étude doivent être répétés périodiquement jusqu'à réversion de la toxicité ou jusqu'à ce quelle soit présumée irréversible.

7.2.3 Chirurgie

- **Congélation tumorale**

Au moment de la chirurgie mammaire, une congélation tumorale pour analyse translationnelle sera également prévue.

- **Evaluation ypTNM**

7.2.4 Bilan de suivi post chirurgie

Les patients seront revus après la chirurgie pour organiser la mise en route éventuelle de la radiothérapie, l'hormonothérapie et/ou la poursuite du trastuzumab.
Les visites de suivi se feront pendant toute la durée de l'étude selon les habitudes des centres (examen clinique, biologique, radiologique…).
Un ECG et une échographie cardiaque seront réalisés 12 mois après le début du traitement

8. EVALUATION DES EVENEMENTS INDESIRABLES GRAVES

8.1 Définition

Est considéré comme un événement indésirable grave (EIG) tout événement :

- Entraînant le décès,
- Mettant en jeu le pronostic vital,
- Entraînant une hospitalisation (≥ 24h) ou une prolongation d'hospitalisation existante,
- Provoquant une invalidité permanente ou une incapacité temporaire grave,
- L'apparition d'un autre cancer,
- Provoquant une anomalie congénitale, une malformation fœtale ou un avortement,
- Médicalement significatif
- Un surdosage médicamenteux (accidentel ou intentionnel),
- Clinique indésirable ou résultat de laboratoire à caractère grave ou considéré comme tel par l'investigateur (avec mention de leur sévérité)

N'est pas considéré comme un événement indésirable grave :

- Une hospitalisation < 24 heures
- Une hospitalisation ou intervention chirurgicale programmée antérieurement à l'inclusion dans l'étude (hospitalisation pour ablation de fils, redons et reconstruction mammaire …) ainsi que toutes les pratiques médicales qui s'inscrivent dans le cadre de cette recherche (hospitalisation pour pose d'un site implantable ou de cathéter…) et dans la mesure où aucune complication n'est observée.
- Toute hospitalisation et tout décès en relation avec la progression du cancer et survenant au-delà de la période de notification définie dans le protocole.

8.2 Définition d'un événement indésirable grave attendu (EIG-A)

Un EIG-A est un événement déjà mentionné dans la version la plus récente de la brochure investigateur ou dans le résumé des caractéristiques du produit (RCP) pour les médicaments ayant déjà une autorisation de mise sur le marché (AMM). Cette définition s'applique également au médicament de l'essai lorsqu'il est administré pour une même population hors indication de l'AMM.

8.3 Définition d'un événement indésirable grave inattendu (EIG-I)

Un EIG-I est un événement non mentionné ou différent par sa nature, son intensité, son évolution par rapport à la brochure investigateur ou au résumé des caractéristiques du produit (RCP) pour les médicaments ayant une autorisation de mise sur le marché (AMM).

8.4 Critère d'intensité

Le critère d'intensité ne doit pas être confondu avec le critère de gravité qui sert de guide pour définir les obligations de déclaration.

L'intensité des événements sera estimée selon la classification CTC-AE V3.0 (toxicité de grade 1 à 5).
L'intensité des événements indésirables non listés dans cette classification sera appréciée selon les qualificatifs suivants :
- Légère (grade 1) : n'affecte pas l'activité quotidienne habituelle du patient
- Modérée (grade 2) : perturbe l'activité quotidienne du patient
- Sévère (grade 3) : empêche l'activité quotidienne habituelle du patient

- Très Sévère (grade 4) : impose des mesures de réanimation/menace le pronostic vital
- Décès (grade 5)

8.5 Période de recueil des évènements indésirables graves

Tout événement indésirable grave survenant jusque dans les 30 jours après la chirurgie doit être notifié par l'investigateur au promoteur.
Au-delà de cette période, seuls les EIG susceptibles d'être dus à la recherche doivent être déclarés au promoteur dès lors qu'aucune autre cause que la recherche ne peut raisonnablement lui être attribuée.

8.6 Conduite à tenir en cas d'événement indésirable grave

La déclaration se fait dans les 24 heures au Bureau d'Etudes Cliniques par envoi par fax ou par mail de la fiche de notification d'un événement indésirable grave.

Bureau d'Etudes Cliniques
Téléphone : 04.91.22.37.78
Fax : 04.91.22.36.01
e-mail : bec@marseille.fnclcc.fr

L'investigateur notera pour chaque événement :
- Sa description, aussi clairement que possible selon la terminologie médicale,
- Si l'événement est attendu ou inattendu,
- Les dates de début et de fin de l'événement,
- Les mesures entreprises et la nécessité ou non d'un traitement correcteur,
- Si le traitement de l'essai a été interrompu,
- Son évolution, en cas d'événement non fatal, l'évolution devra être suivie jusqu'à la guérison ou le retour à l'état antérieur ou à la stabilisation d'éventuelles séquelles,
- La relation de causalité entre cet événement et le traitement à l'essai,
- La relation de causalité avec les médicaments de l'essai, la pathologie traitée, une autre pathologie ou un autre traitement.

Un rapport de suivi est ensuite adressé au promoteur pour documenter l'évolution de l'événement en remplissant les différents suivis du formulaire de déclaration initiale de recueil d'un EIG.

9. STATISTIQUES

9.1 Analyse statistique des données

Les données qualitatives seront présentées sous forme d'effectifs et de pourcentages, les données continues seront résumées par la moyenne, l'écart type, la médiane et les valeurs extrêmes. Des intervalles de confiance à 95% seront estimés pour le critère principal et les critères secondaires.

La différence entre le pourcentage de cellules souches après traitement et le même pourcentage avant traitement sera calculée pour tous les patients. Dans le bras expérimental, un test d'équivalence sera utilisé pour valider l'hypothèse que cette différence ne dépasse pas 5%.
Les analyses statistiques des études ancillaires sont détaillées en annexe 4.

9.2 Nombre de patients et durée de l'étude

L'objectif est de montrer, dans le bras expérimental, que la différence d'expression d'ALDH1+ exprimé en pourcentage de cellules (après traitement «moins» avant traitement) est inférieure à 5. Un test d'équivalence sera utilisé sous les hypothèses suivantes :
• Différence d'expression nulle en moyenne
• Variabilité de la différence σ = 13 (estimation basée sur les données de la littérature et supposant une corrélation intra patient ρ=0.4)
• Risque alpha de 0.05, puissance de 0.8
• δ= 5 (écart maximum toléré)

Il est nécessaire d'inclure 42 patients dans le bras expérimental. En considérant un maximum de 20% d'échantillons non évaluable, 50 patients seront inclus dans le bras expérimental. Le bras contrôle, nécessaire pour vérifier l'hypothèse d'un triplement de l'expression d'ALDH1+, sera attribué par randomisation selon le ratio 1:2.
Au total, 75 patients seront inclus dans l'étude, répartis sur 5 centres de recherche et randomisés selon un ratio 1:2 entre le bras contrôle et le bras expérimental (50 patients dans le bras expérimental, 25 patients dans le bras contrôle).

La période d'inclusion sera de 3 ans.

La fin de l'étude est définie comme 5 ans après l'inclusion du dernier patient.

9.3 Degré de signification statistique prévu

Tous les résultats faisant appel à un estimateur seront présentés avec un intervalle de confiance à 95%. Les tests seront bilatéraux au seuil de 5%.

9.4 Critères statistiques d'arrêt de la recherche

Aucune analyse intermédiaire n'est planifiée avant la fin des inclusions.
Aucun critère statistique d'arrêt de la recherche n'est prévu dans cette étude.

9.5 Méthode de prise en compte des données manquantes

Aucune méthode d'imputation des données manquantes ne sera utilisée dans cette étude.

9.6 Gestion des modifications apportées au plan d'analyse de la stratégie initiale.

Toute modification apportée au plan d'analyse de la stratégie initiale fera l'objet d'une demande écrite au responsable du bureau d'études cliniques de l'Institut Paoli-Calmettes.

9.7 Choix des personnes à inclure dans les analyses.

Tous les patients inclus dans l'étude et ayant pu faire l'objet d'une mesure du nombre de cellules avant et après traitement seront inclus dans les analyses.

10. DROITS D'ACCÈS AUX DONNÉES

L'investigateur s'engage, pour lui-même et pour toutes les personnes amenées à suivre le déroulement de l'essai, à garantir la confidentialité de toutes les informations fournies par l'Institut Paoli-Calmettes jusqu'à la publication des résultats de l'essai. Cette obligation de confidentialité ne s'appliquera pas aux renseignements que l'investigateur sera amené à communiquer aux patients dans le cadre de leur participation à l'essai ni aux informations déjà publiées.

L'investigateur s'engage à ne pas publier, divulguer ou utiliser, de quelque façon que ce soit, directement ou indirectement, les informations scientifiques ou techniques en relation avec l'essai.

Néanmoins, conformément à l'article R 5121-13 du Code de la Santé Publique, le centre et l'investigateur pourront donner des informations relatives à l'essai :
- au ministre chargé de la santé,
- aux médecins inspecteurs de santé publique,
- aux pharmaciens inspecteurs de santé publique,
- au Directeur Général et aux inspecteurs de l'Afssaps.

L'essai ne pourra faire l'objet d'aucun commentaire écrit ou oral sans l'accord du promoteur ; l'ensemble des informations communiquées ou obtenues pendant la réalisation de l'essai appartenant de plein droit au promoteur qui pourra librement en disposer.

11. CONTROLE ET ASSURANCE QUALITE

Le promoteur est responsable de la mise en place d'un système d'assurance qualité décrit dans les procédures institutionnelles afin que l'essai soit réalisé conformément au protocole et aux Bonnes Pratiques Cliniques.

12. CONSIDERATIONS ETHIQUES ET REGLEMENTAIRES

L'essai clinique doit être conduit conformément :

- aux principes éthiques de la déclaration d'Helsinki de 1964, révisée
- aux Bonnes Pratiques Cliniques de la décision de Novembre 2006,
- à la Directive Européenne (2001/20/CE) sur la conduite des essais cliniques,
- à la loi Huriet (n°88-1138) du 20 décembre 1988 relative à la Protection des Personnes se prêtant à la Recherche Biomédicale et modifiée par la loi de Santé Publique (n° 2004-806) du 9 août 2004,
- à la loi n° 2004-801 du 6 août 2004 relative à la protection des personnes physiques à l'égard des traitements de données à caractère personnel, et modifiant la loi n° 78-17 du 6 janvier 1978 relative à l'informatique, aux fichiers et aux libertés,
- à la loi n° 2004-800 du 6 août 2004 relative à la bioéthique.

12.1 Comité de Protection des Personnes (CPP)

Le promoteur s'engage à soumettre le projet d'étude à l'avis préalable d'un Comité de Protection des Personnes (CPP). Les informations communiquées portent d'une part sur les modalités et la nature de la recherche et d'autre part, sur les garanties prévues pour les patients participant à cet essai.

12.2 Déclaration aux autorités compétentes

Le présent protocole fera l'objet d'une demande d'autorisation auprès de l'Afssaps.

12.3 Information et Consentement des participants

Préalablement à la réalisation d'une recherche biomédicale sur une personne, le consentement libre, éclairé et exprès de celle-ci doit être recueilli après qu'elle ait été informée, par l'investigateur ou son représentant, de l'objectif de la recherche, du déroulement et de la durée de l'étude, des bénéfices, risques potentiels et contraintes de l'essai ainsi que de la nature du produit étudié et de l'avis donné par le CPP et de l'autorisation de l'AFSSAPS.

Le formulaire de consentement sera daté et signé personnellement par le patient et l'investigateur (un exemplaire archivé par l'investigateur, un autre remis au patient ou à son représentant légal).

Le formulaire d'information et de consentement éclairé (annexe 8) destiné au patient doit être associé sur un même document afin d'éviter tout risque de contestation sur le contenu de l'information donnée.

12.4 Responsabilités des investigateurs

L'investigateur de chaque établissement concerné s'engage à conduire l'essai clinique conformément au protocole qui a été approuvé par le CPP et l'AFSSAPS. L'investigateur ne doit apporter aucune modification au protocole sans l'autorisation du promoteur.

Il est de la responsabilité de l'investigateur responsable de l'essai dans l'établissement :
- de fournir au promoteur son curriculum vitae ainsi que ceux des co-investigateurs,
- d'identifier les membres de son équipe qui participent à l'essai et de définir leurs responsabilités,
- de démarrer le recrutement des patients après autorisation du promoteur,
- d'essayer d'inclure le nombre requis de patients dans les limites de la période de recrutement établie.

Il est de la responsabilité de chaque investigateur :
- de recueillir le consentement éclairé daté et signé personnellement par le patient avant toute procédure de sélection spécifique à l'essai,
- de compléter régulièrement les cahiers d'observation (CRF) pour chacun des patients inclus dans l'essai et de laisser à l'Assistant de Recherche Clinique (ARC) un accès direct aux documents-source afin que ce dernier puisse valider les données du CRF,
- de corriger, de signer et de dater les corrections des CRF pour chacun des patients inclus dans l'étude,
- de notifier au promoteur les événements indésirables graves dans les délais exigés
- d'accepter les visites régulières de l'ARC et éventuellement celles des auditeurs mandatés par le promoteur ou des inspecteurs des autorités de tutelle.

Toute la documentation relative à l'étude (protocole, consentements, cahiers d'observation, dossier investigateur, etc.…), ainsi que les documents originaux (résultats de laboratoire, radiologies, comptes-rendus de consultations, rapports d'examens cliniques pratiqués, etc.) doivent être détenus dans un lieu sûr et considérés comme du matériel confidentiel. L'archivage des données sera sous la responsabilité de l'investigateur et selon la législation en vigueur.
Ce dernier devra conserver les données ainsi qu'une liste d'identification des patients pendant une durée minimale de 15 ans après la fin de l'étude.

12.5 Responsabilités du promoteur

Il est de la responsabilité du promoteur de :
- souscrire une assurance pour couvrir sa responsabilité civile en cas de conséquences dommageables de la recherche pour la personne qui s'y prête,
- communiquer aux investigateurs toutes les informations nécessaires pour la conduite de la recherche,
- demander l'avis du CPP,
- demander l'autorisation à l'AFSSAPS
- d'informer les Directeurs et les Pharmaciens des établissements de santé
- informer l'AFSSAPS de tout événement grave susceptible d'être dû à la recherche ou de l'arrêt prématuré de celle-ci à partir du moment où il en a été informé

Le promoteur doit assurer l'archivage des documents essentiels sur la conduite de l'étude dans des conditions assurant leur sécurité, pendant la durée minimale prévue par les BPC, soit 15 ans après la fin de la recherche.

12.6 Comité de Patients

Le Comité de Patients de la Ligue Nationale Contre le Cancer s'engage dans le cadre des essais cliniques en cancérologie promus par l'IPC à relire le protocole et à proposer des améliorations portant notamment sur la qualité de la lettre d'information, la mise à disposition d'un plan de traitement et de surveillance, la suggestion de mesures visant à améliorer le confort des patients et selon la charte qui a été définie entre le Comité de Patients (LNCC) et le BECT de la FNCLCC.

13. TRAITEMENT DES DONNÉES ET CONSERVATION DES DOCUMENTS ET DES DONNÉES RELATIVES À LA RECHERCHE

Le traitement des données de l'essai est assuré par le Centre de Traitement des Données sous la responsabilité du Dr Dominique GENRE; les données restant la propriété de l'Institut Paoli-Calmettes, promoteur de la recherche.

Le logiciel de traitement des données est le logiciel Capture System.

Conformément à la révision de la loi informatique et liberté du 06 août 2004 et à son décret d'application, l'Institut Paoli-Calmettes s'est engagé à suivre la méthodologie de référence MR001 de la Commission Nationale de l'Informatique et des Libertés.

Toute la documentation relative à l'essai (protocole, consentements, cahiers d'observation, dossier investigateur, etc…), ainsi que les documents originaux (résultats de laboratoire, radiologies, comptes-rendus de consultations, rapports d'examens cliniques, etc.) est considérée comme confidentielle et doit être détenue dans un lieu sûr. L'investigateur principal devra conserver les données ainsi qu'une liste d'identification des patients pendant une durée minimale de 15 ans après la fin de l'étude.

14. FINANCEMENT ET ASSURANCE

Le bevacizumab sera fourni par le promoteur.
L'IPC souscrit une assurance de recherches biomédicales, conformément à la législation en vigueur, auprès de la société SHAM (18 rue Edouard Rochet – 69372 LYON CEDEX 08).

15. RÈGLES RELATIVES À LA PUBLICATION

Les résultats de cet essai sont la propriété exclusive de l'Institut Paoli-Calmettes qui pourra les exploiter librement. Toutes les informations résultant de cet essai sont considérées comme confidentielles, au moins jusqu'à ce que l'analyse appropriée et le contrôle par le promoteur et l'investigateur principal soient achevés. Les résultats doivent être publiés ou présentés en collaboration avec le promoteur.

Toutes publications, abstracts ou présentations comprenant les résultats de l'essai doivent être soumis pour approbation au promoteur (IPC).

Par ailleurs, toutes communications, manuscrits ou présentations doivent comporter une rubrique qui mentionne impérativement l'IPC et l'UMR 599 ainsi que les organismes qui ont supporté financièrement la recherche.

L'investigateur coordonnateur de l'essai sera le signataire principal (1er ou dernier auteur).

Les investigateurs suivants seront cités en fonction du nombre de patients recrutés dans le cadre d'essais multicentriques, ou en fonction de leur implication dans le protocole et/ou la pathologie. Le statisticien de l'essai ainsi qu'un membre du BEC seront également cités.

De même, les publications des résultats annexes (étude biologique) comporteront le nom de la personne ayant réalisé le travail annexe ainsi que les noms de toutes les autres personnes concernées par ce travail annexe.

REFERENCES

1. Reya T, Morrison SJ, Clarke MF, et al: Stem cells, cancer, and cancer stem cells. Nature 414:105-11, 2001
2. Bonnet D, Dick JE: Human acute myeloid leukemia is organized as a hierarchy that originates from a primitive hematopoietic cell. Nat Med 3:730-7, 1997
3. Al-Hajj M, Wicha MS, Benito-Hernandez A, et al: Prospective identification of tumorigenic breast cancer cells. Proc Natl Acad Sci U S A 100:3983-8, 2003
4. Ginestier C, Hur MH, Charafe-Jauffret E, et al: ALDH1 is a marker of normal and malignant human mammary stem cells and a predictor of poor clinical outcome. Cell Stem Cell 1:555-67, 2007
5. Al-Hajj M, Becker MW, Wicha M, et al: Therapeutic implications of cancer stem cells. Curr Opin Genet Dev 14:43-7, 2004
6. Li F, Tiede B, Massague J, et al: Beyond tumorigenesis: cancer stem cells in metastasis. Cell Res 17:3-14, 2007
7. Phillips TM, McBride WH, Pajonk F: The response of CD24(-/low)/CD44+ breast cancer-initiating cells to radiation. J Natl Cancer Inst 98:1777-85, 2006
8. Wicha MS, Liu S, Dontu G: Cancer stem cells: an old idea--a paradigm shift. Cancer Res 66:1883-90; discussion 1895-6, 2006
9. Weidner N, Folkman J, Pozza F, et al: Tumor angiogenesis: a new significant and independent prognostic indicator in early-stage breast carcinoma. J Natl Cancer Inst 84:1875-87, 1992
10. Ferrara N, Davis-Smyth T: The biology of vascular endothelial growth factor. Endocr Rev 18:4-25, 1997
11. Liu CD, Tilch L, Kwan D, et al: Vascular endothelial growth factor is increased in ascites from metastatic pancreatic cancer. J Surg Res 102:31-4, 2002
12. Itakura J, Ishiwata T, Friess H, et al: Enhanced expression of vascular endothelial growth factor in human pancreatic cancer correlates with local disease progression. Clin Cancer Res 3:1309-16, 1997
13. Yoshiji H, Gomez DE, Shibuya M, et al: Expression of vascular endothelial growth factor, its receptor, and other angiogenic factors in human breast cancer. Cancer Res 56:2013-6, 1996
14. Fontanini G, Vignati S, Lucchi M, et al: Neoangiogenesis and p53 protein in lung cancer: their prognostic role and their relation with vascular endothelial growth factor (VEGF) expression. Br J Cancer 75:1295-301, 1997
15. Obermair A, Kucera E, Mayerhofer K, et al: Vascular endothelial growth factor (VEGF) in human breast cancer: correlation with disease-free survival. Int J Cancer 74:455-8, 1997
16. Linderholm B, Tavelin B, Grankvist K, et al: Vascular endothelial growth factor is of high prognostic value in node-negative breast carcinoma. J Clin Oncol 16:3121-8, 1998
17. Linderholm B, Grankvist K, Wilking N, et al: Correlation of vascular endothelial growth factor content with recurrences, survival, and first relapse site in primary node-positive breast carcinoma after adjuvant treatment. J Clin Oncol 18:1423-31, 2000
18. Heffelfinger SC, Miller MA, Yassin R, et al: Angiogenic growth factors in preinvasive breast disease. Clin Cancer Res 5:2867-76, 1999
19. Salven P, Perhoniemi V, Tykka H, et al: Serum VEGF levels in women with a benign breast tumor or breast cancer. Breast Cancer Res Treat 53:161-6, 1999
20. Takahashi Y, Tucker SL, Kitadai Y, et al: Vessel counts and expression of vascular endothelial growth factor as prognostic factors in node-negative colon cancer. Arch Surg 132:541-6, 1997

21. Jain RK: Normalizing tumor vasculature with anti-angiogenic therapy: A new paradigm for combination therapy. Nat Med 7:987-989, 2001

22. Bao S, Wu Q, Sathornsumetee S, et al: Stem cell-like glioma cells promote tumor angiogenesis through vascular endothelial growth factor. Cancer Res 66:7843-8, 2006

23. Rich JN: Cancer stem cells in radiation resistance. Cancer Res 67:8980-4, 2007

24. Vredenburgh JJ, Desjardins A, Herndon JE, 2nd, et al: Phase II trial of bevacizumab and irinotecan in recurrent malignant glioma. Clin Cancer Res 13:1253-9, 2007

25. Vredenburgh JJ, Desjardins A, Herndon JE, 2nd, et al: Bevacizumab plus irinotecan in recurrent glioblastoma multiforme. J Clin Oncol 25:4722-9, 2007

26. Sathornsumetee S, Cao Y, Marcello JE, et al: Tumor angiogenic and hypoxic profiles predict radiographic response and survival in malignant astrocytoma patients treated with bevacizumab and irinotecan. J Clin Oncol 26:271-8, 2008

27. Li X, Lewis MT, Huang J, et al: Intrinsic resistance of tumorigenic breast cancer cells to chemotherapy. J Natl Cancer Inst 100:672-9, 2008

28. Hortobagyi GN, Ames FC, Buzdar AU, et al: Management of stage III primary breast cancer with primary chemotherapy, surgery, and radiation therapy. Cancer 62:2507-16, 1988

29. Jacquillat C, Weil M, Baillet F, et al: Results of neoadjuvant chemotherapy and radiation therapy in the breast-conserving treatment of 250 patients with all stages of infiltrative breast cancer. Cancer 66:119-29, 1990

30. Viens P, Penault-Llorca F, Jacquemier J, et al: High-dose chemotherapy and haematopoietic stem cell transplantation for inflammatory breast cancer: pathologic response and outcome. Bone Marrow Transplant 21:249-54, 1998

31. Bonadonna G, Valagussa P, Brambilla C, et al: Primary chemotherapy in operable breast cancer: eight-year experience at the Milan Cancer Institute. J Clin Oncol 16:93-100, 1998

32. Smith IE, Jones AL, O'Brien ME, et al: Primary medical (neo-adjuvant) chemotherapy for operable breast cancer. Eur J Cancer 29A:1796-9, 1993

33. Forrest AP, Levack PA, Chetty U, et al: A human tumour model. Lancet 2:840-2, 1986

34. Anderson ED, Forrest AP, Hawkins RA, et al: Primary systemic therapy for operable breast cancer. Br J Cancer 63:561-6, 1991

35. Cameron DA, Anderson ED, Levack P, et al: Primary systemic therapy for operable breast cancer--10-year survival data after chemotherapy and hormone therapy. Br J Cancer 76:1099-105, 1997

36. Belembaogo E, Feillel V, Chollet P, et al: Neoadjuvant chemotherapy in 126 operable breast cancers. Eur J Cancer 28A:896-900, 1992

37. Smith IE, Walsh G, Jones A, et al: High complete remission rates with primary neoadjuvant infusional chemotherapy for large early breast cancer. J Clin Oncol 13:424-9, 1995

38. Calais G, Berger C, Descamps P, et al: Conservative treatment feasibility with induction chemotherapy, surgery, and radiotherapy for patients with breast carcinoma larger than 3 cm. Cancer 74:1283-8, 1994

39. Brain E, Garrino C, Misset JL, et al: Long-term prognostic and predictive factors in 107 stage II/III breast cancer patients treated with anthracycline-based neoadjuvant chemotherapy. Br J Cancer 75:1360-7, 1997

40. Bonadonna G, Veronesi U, Brambilla C, et al: Primary chemotherapy to avoid mastectomy in tumors with diameters of three centimeters or more. J Natl Cancer Inst 82:1539-45, 1990

41. Rouzier R, Mathieu MC, Sideris L, et al: Breast-conserving surgery after neoadjuvant anthracycline-based chemotherapy for large breast tumors. Cancer 101:918-25, 2004

42. Amat S, Bougnoux P, Penault-Llorca F, et al: Neoadjuvant docetaxel for operable breast cancer induces a high pathological response and breast-conservation rate. Br J Cancer 88:1339-45, 2003

43. Gradishar WJ: Docetaxel as neoadjuvant chemotherapy in patients with stage III breast cancer. Oncology (Williston Park) 11:15-8, 1997

44. Miller KD, McCaskill-Stevens W, Sisk J, et al: Combination versus sequential doxorubicin and docetaxel as primary chemotherapy for breast cancer: A randomized pilot trial of the Hoosier Oncology Group. J Clin Oncol 17:3033-7, 1999

45. von Minckwitz G, Raab G, Caputo A, et al: Doxorubicin with cyclophosphamide followed by docetaxel every 21 days compared with doxorubicin and docetaxel every 14 days as preoperative treatment in operable breast cancer: the GEPARDUO study of the German Breast Group. J Clin Oncol 23:2676-85, 2005

46. Kuerer HM, Newman LA, Smith TL, et al: Clinical course of breast cancer patients with complete pathologic primary tumor and axillary lymph node response to doxorubicin-based neoadjuvant chemotherapy. J Clin Oncol 17:460-9, 1999

47. Bear HD, Anderson S, Brown A, et al: The effect on tumor response of adding sequential preoperative docetaxel to preoperative doxorubicin and cyclophosphamide: preliminary results from National Surgical Adjuvant Breast and Bowel Project Protocol B-27. J Clin Oncol 21:4165-74, 2003

48. Watatani M, Ueda K, Daito K, et al: Clinical experience of weekly paclitaxel-based treatment as preoperative chemotherapy for patients with primary breast cancer. Breast Cancer 11:187-93, 2004

49. Dieras V, Fumoleau P, Romieu G, et al: Randomized parallel study of doxorubicin plus paclitaxel and doxorubicin plus cyclophosphamide as neoadjuvant treatment of patients with breast cancer. J Clin Oncol 22:4958-65, 2004

50. Smith IC, Heys SD, Hutcheon AW, et al: Neoadjuvant chemotherapy in breast cancer: significantly enhanced response with docetaxel. J Clin Oncol 20:1456-66, 2002

51. Hutcheon AW, Heys SD, Sarkar TK: Neoadjuvant docetaxel in locally advanced breast cancer. Breast Cancer Res Treat 79 Suppl 1:S19-24, 2003

52. Estevez LG, Cuevas JM, Anton A, et al: Weekly docetaxel as neoadjuvant chemotherapy for stage II and III breast cancer: efficacy and correlation with biological markers in a phase II, multicenter study. Clin Cancer Res 9:686-92, 2003

53. Mauriac L, MacGrogan G, Avril A, et al: Neoadjuvant chemotherapy for operable breast carcinoma larger than 3 cm: a unicentre randomized trial with a 124-month median follow-up. Institut Bergonie Bordeaux Groupe Sein (IBBGS). Ann Oncol 10:47-52, 1999

54. Mauriac L, Durand M, Avril A, et al: Effects of primary chemotherapy in conservative treatment of breast cancer patients with operable tumors larger than 3 cm. Results of a randomized trial in a single centre. Ann Oncol 2:347-54, 1991

55. Scholl SM, Fourquet A, Asselain B, et al: Neoadjuvant versus adjuvant chemotherapy in premenopausal patients with tumours considered too large for breast conserving surgery: preliminary results of a randomised trial: S6. Eur J Cancer 30A:645-52, 1994

56. Semiglazov VF, Topuzov EE, Bavli JL, et al: Primary (neoadjuvant) chemotherapy and radiotherapy compared with primary radiotherapy alone in stage IIb-IIIa breast cancer. Ann Oncol 5:591-5, 1994

57. Powles TJ, Hickish TF, Makris A, et al: Randomized trial of chemoendocrine therapy started before or after surgery for treatment of primary breast cancer. J Clin Oncol 13:547-52, 1995

58. van der Hage JA, van de Velde CJ, Julien JP, et al: Preoperative chemotherapy in primary operable breast cancer: results from the European Organization for Research and Treatment of Cancer trial 10902. J Clin Oncol 19:4224-37, 2001

59. Mauri D, Pavlidis N, Ioannidis JP: Neoadjuvant versus adjuvant systemic treatment in breast cancer: a meta-analysis. J Natl Cancer Inst 97:188-94, 2005

60. Coudert BP, Largillier R, Arnould L, et al: Multicenter phase II trial of neoadjuvant therapy with trastuzumab, docetaxel, and carboplatin for human epidermal growth factor receptor-2-overexpressing stage II or III breast cancer: results of the GETN(A)-1 trial. J Clin Oncol 25:2678-84, 2007

61. Coudert BP, Arnould L, Moreau L, et al: Pre-operative systemic (neo-adjuvant) therapy with trastuzumab and docetaxel for HER2-overexpressing stage II or III breast cancer: results of a multicenter phase II trial. Ann Oncol 17:409-14, 2006

62. Buzdar AU, Ibrahim NK, Francis D, et al: Significantly Higher Pathologic Complete Remission Rate After Neoadjuvant Therapy With Trastuzumab, Paclitaxel, and Epirubicin Chemotherapy: Results of a Randomized Trial in Human Epidermal Growth Factor Receptor 2-Positive Operable Breast Cancer. J Clin Oncol 23:3676-3685, 2005

63. Borgstrom P, Hillan KJ, Sriramarao P, et al: Complete inhibition of angiogenesis and growth of microtumors by anti-vascular endothelial growth factor neutralizing antibody: novel concepts of angiostatic therapy from intravital videomicroscopy. Cancer Res 56:4032-9, 1996

64. Sweeney CJ, Miller KD, Sissons SE, et al: The antiangiogenic property of docetaxel is synergistic with a recombinant humanized monoclonal antibody against vascular endothelial growth factor or 2-methoxyestradiol but antagonized by endothelial growth factors. Cancer Res 61:3369-72, 2001

65. Kerbel RS: Tumor angiogenesis. N Engl J Med 358:2039-49, 2008

66. Lee CG, Heijn M, di Tomaso E, et al: Anti-Vascular endothelial growth factor treatment augments tumor radiation response under normoxic or hypoxic conditions. Cancer Res 60:5565-70, 2000

67. Tong RT, Boucher Y, Kozin SV, et al: Vascular normalization by vascular endothelial growth factor receptor 2 blockade induces a pressure gradient across the vasculature and improves drug penetration in tumors. Cancer Res 64:3731-6, 2004

68. Shaked Y, Ciarrocchi A, Franco M, et al: Therapy-induced acute recruitment of circulating endothelial progenitor cells to tumors. Science 313:1785-7, 2006

69. Lee TH, Seng S, Sekine M, et al: Vascular endothelial growth factor mediates intracrine survival in human breast carcinoma cells through internally expressed VEGFR1/FLT1. PLoS Med 4:e186, 2007

70. Cobleigh MA, Langmuir VK, Sledge GW, et al: A phase I/II dose-escalation trial of bevacizumab in previously treated metastatic breast cancer. Semin Oncol 30:117-24, 2003

71. Miller KD, Chap LI, Holmes FA, et al: Randomized phase III trial of capecitabine compared with bevacizumab plus capecitabine in patients with previously treated metastatic breast cancer. J Clin Oncol 23:792-9, 2005

72. Miller K, Wang M, Gralow J, et al: Paclitaxel plus bevacizumab versus paclitaxel alone for metastatic breast cancer. N Engl J Med 357:2666-76, 2007

73. Miles D, Chan A, Romieu G, et al: Randomized, double-blind, placebo-controlled, phase III study of bevacizumab with docetaxel or docetaxel with placebo as first-line therapy for patients with locally recurrent or metastatic breast cancer (mBC): AVADO. J Clin Oncol (Meeting Abstracts) 26:LBA1011-, 2008

74. Wedam SB, Low JA, Yang SX, et al: Antiangiogenic and antitumor effects of bevacizumab in patients with inflammatory and locally advanced breast cancer. J Clin Oncol 24:769-77, 2006

75. Thukral A, Thomasson DM, Chow CK, et al: Inflammatory breast cancer: dynamic contrast-enhanced MR in patients receiving bevacizumab--initial experience. Radiology 244:727-35, 2007

76. Mayer EL, Miller KD, Rugo HS, et al: A pilot study of adjuvant bevacizumab and chemotherapy after neoadjuvant chemotherapy for high-risk breast cancer. J Clin Oncol (Meeting Abstracts) 26:519-, 2008

77. Greil R, Moik M, Reitsamer R, et al: Efficacy of neoadjuvant bevacizumab (Av), capecitabine (X), and docetaxel (T) for invasive breast cancer (BC): Phase II result. J Clin Oncol (Meeting Abstracts) 26:14575-, 2008

78. Morris PG, Dickler MN, McArthur HL, et al: Dose-dense (dd) doxorubicin-cyclophosphamide (AC) X 4 and short-term changes in left ventricular ejection fraction (LVEF) alone or with bevacizumab (B) in patients (pts) with early stage breast cancer (BC). J Clin Oncol (Meeting Abstracts) 26:637-, 2008

79. Liu G, Yuan X, Zeng Z, et al: Analysis of gene expression and chemoresistance of CD133+ cancer stem cells in glioblastoma. Mol Cancer 5:67, 2006

80. Plasschaert SL, van der Kolk DM, de Bont ES, et al: The role of breast cancer resistance protein in acute lymphoblastic leukemia. Clin Cancer Res 9:5171-7, 2003

81. Salmaggi A, Boiardi A, Gelati M, et al: Glioblastoma-derived tumorospheres identify a population of tumor stem-like cells with angiogenic potential and enhanced multidrug resistance phenotype. Glia 54:850-60, 2006

82. Ginestier C, Hur MH, Charafe-Jauffret E, et al: ALDH1 Is a Marker of Normal and Malignant Human Mammary Stem Cells and a Predictor of Poor Clinical Outcome. Cell Stem Cell 1:555-567, 2007

83. Calabrese C, Poppleton H, Kocak M, et al: A Perivascular Niche for Brain Tumor Stem Cells. Cancer Cell 11:69-82, 2007

84. Folkins C, Man S, Xu P, et al: Anticancer Therapies Combining Antiangiogenic and Tumor Cell Cytotoxic Effects Reduce the Tumor Stem-Like Cell Fraction in Glioma Xenograft Tumors. Cancer Res 67:3560-3564, 2007

ANNEXES

ANNEXE 1 : TABLEAU RECAPITULATIF DES INVESTIGATIONS

ANNEXE 2 : EVALUATION DE L'ETAT GENERAL EN FONCTION DE LA CLASSIFICATION DE KARNOFSKY (OMS)

ANNEXE 3 : MACROBIOPSIES ET MARQUAGE ALDH1 EN IMMUNOHISTOCHIMIE

ANNEXE 4 : ETUDES ANCILLAIRES

ANNEXE 5 : GESTION DES TOXICITÉS INDUITES PAR LE BEVACIZUMAB

ANNEXE 6 : ECHELLE DE TOXICITE NCI CTC-AE v3.0

ANNEXE 7 : CLASSIFICATION DE LA NYHA

ANNEXE 8 : RÉSUMÉ DES CARACTÉRISTIQUES DES PRODUITS

ANNEXE 1 : TABLEAU RECAPITULATIF DES INVESTIGATIONS

	Bilan inclusion	Bilan de suivi en cours de traitement									
**TRAITEMENT**	**J-28**	**C1 J1**	**C2 J22**	**C3 J43**	**C4 J64**	**C5 J85**	**C6 J106**	**C7 J127**	**C8 J148**	**Avant Chir**	**M12**
Critères d'inclusion / non inclusion	X										
Antécédents/histoire de la maladie	X										
Consentement éclairé signé	X										
**EXAMEN CLINIQUE**											
Taille, Poids, Surface corporelle, Karnofsky	X					X(poids)					
Examen clinique, FC, TA	X	X	X	X	X	X	X	X	X		
Toxicités (CTCAE et NYHA pour toxicité cardiaque) / traitements concomitants			X	X	X	X	X	X	X		
**EXAMEN PARACLINIQUE**											
Echo-mammographie	X										
Scanner thoracique et abdominal/pelvien ou TEP-TDM	X										
Scintigraphie osseuse (sauf si TEP-TDM)	X										
Echographie mammaire avec injection de produit de contraste (étude ancillaire)	*X*		*X*			*X*				*X*	
ECG, Echographie cardiaque (FEVG)	X					X				X	X
**BILAN BIOLOGIQUE**											
NFS, Plaquettes, TP, INR, TCA	X	X	X	X	X	X	X	X	X	X	
Ionogramme, Calcémie, LDH, phosphates, protéines, albumine,	X	X	X	X	X	X	X	X	X	X	
Bilan hépatique : bilirubine, ALAT, ASAT, PAL, GGT	X	X	X	X	X	X	X	X	X	X	
Bilan rénal (créatinine, urée), bandelette urinaire (protéinurie)	X	X	X	X	X	X	X	X	X	X	
Marqueurs tumoraux (ACE, CA 15-3)	X										
~~2 tubes~~ 1 tube de 10 ml de sang total Veridex® pour traitement immédiat (CTC ~~et CEC~~)<br>2 tubes EDTA et 2 tubes secs de 5 ml pour congélation (études translationnelles)	X<br>X		X<br>X							X<br>X	
**EXAMEN HISTOLOGIQUE**											
Biopsie tumorale ou macrobiopsie : fixation (IHC pour ALDH1+), congélation (transcriptomique/protéomique) et dissociation fraîche (à l'IPC pour xenogreffe),	X (typage HER2 et RE, RP)					X				X	

ANNEXE 2 : EVALUATION DE L'ETAT GENERAL EN FONCTION DE LA CLASSIFICATION DE KARNOFSKY (OMS)

ETAT GENERAL<br><br>KARNOFSKY	ECHELLE		ETAT GENERAL<br><br>ECOG-ZUBROD/WHO
Normal, pas de plaintes.<br><br>Activité normale. Signes ou symptômes mineurs de la maladie.	100<br><br>90	0	Activité normale, sans restriction.
Activité normale avec efforts.<br><br>Capable de se prendre en charge, mais incapable d'avoir une activité normale ou de travailler.	80<br><br>70	1	Restreint pour des activités physiques importantes mais patient ambulatoire et capable de fournir un travail léger.
Nécessite occasionnellement de l'aide, mais capable de subvenir à la plupart de ses besoins.<br><br>Nécessite aide et soins médicaux fréquents.	60<br><br>50	2	Ambulatoire et capable de se prendre en charge, mais incapable de fournir un travail pendant plus 50% de son temps.
Nécessite soins médicaux et aide importante.<br><br>Sévèrement limité, grabataire. Indication d'hospitalisation, quoique la mort ne soit pas imminente.	40<br><br>30	3	Capacité de prise en charge propre beaucoup plus limitée. Passe plus de 50 % de son temps au lit ou dans une chaise.
Gravement atteint. Hospitalisation nécessaire. Traitement symptomatique nécessaire<br><br>Moribond ; Processus fatal progressant rapidement	20<br><br>10	4	Complètement grabataire. Incapable de se prendre en charge. Le patient reste totalement couché au lit ou sur une chaise.

Référence : Common Toxicity Criteria v2.0 révisée (CTC), publiée le 30 Avril 1999

ANNEXE 3 : MACROBIOPSIES ET MARQUAGE ALDH1 EN IMMUNOHISTOCHIMIE

1. Prise en charge de la biopsie au laboratoire d'anatomie pathologique

- Recueil du matériel biopsique à l'état frais et dans le délai le plus court par rapport à la réalisation du prélèvement
- Réalisation du «tri» des prélèvements : si la taille et la qualité des carottes biopsiques le permettent, mise dans le fixateur d'au minimum une carotte pour le diagnostic histologique et la réalisation des études immunohistochimiques (fixateur recommandé : Formol tamponné 10%) - cryoconservation d'au minimum 2 carottes en cryotubes pour la réalisation d'extraction d'ARN (dans l'azote liquide à -136°C) - 2 carottes pour les études protéomiques (à -80°C)- le reste des carottes disponibles étant techniqué pour marquage ALDEFLUOR après dissociation du tissu frais.

2. Dissociation du tissu frais et marquage ALDEFLUOR

Dissociation tumorale et marquage ALDEFLUOR - tri par FACS

Les échantillons tumoraux provenant de biopsies ou d'exérèse chirurgicale sont découpés en petits fragments millimétriques et dissociés par méthode enzymatique (Dontu et al) afin d'obtenir une suspension unicellulaire. Le kit ALDEFLUOR (StemCell technologies, Durham, NC, USA) est utilisé pour isoler la population qui possède une forte activité enzymatique ALDEFLUOR. Les cellules sont resuspendues dans du tampon ALDEFLUOR contenant le substrat de l'ALDH (BAAA, 1 µmol/l pour 1x106 cellules) et incubées 40 minutes à 37°C. Pour chaque expérience, un échantillon de cellules est marqué dans les mêmes conditions avec 50mmol/L d'inhibiteur d'ALDH, diethylaminobenzaldehyde (DEAB) comme contrôle négatif. Les cellules sont triées sur un cytomètre de flux type FACStarPLUS (Becton Dickinson). La fluorescence de l'ALDEFLUOR est excitée à 488 nm et son émission est recueillie en utilisant un filtre standard fluorescéine isothiocyanate (FITC) à 530/30. Une incubation avec un mix d'anticorps anti-lignage est effectuée et permet d'éliminer contamination stromale et infiltrat inflammatoire. Les fenêtres de tri sont établies en utilisant comme contrôle négatif des cellules colorées au iodure de Propidium pour la viabilité, les cellules marquées à l'ALDEFLUOR traitées au DEAB et les cellules marquées lignage seul.

On pourra rajouter après tri une étape de perméabilisation et marquage avec un anticorps anti-VEGF – puis anticorps secondaire couplé à un fluorochrome afin de quantifier en cytométrie de flux la proportion de cellules ALDE+ ou – qui expriment VEGF.
Cette approche sur tissu frais est une approche complémentaire de l'immunomarquage sur tissu fixé plus large car liée à l'activité enzymatique de différentes isoformes d'ALDH et qui pourra d'ailleurs être évaluée comparativement à cette dernière pour les mêmes tumeurs.

3. Marquage in situ sur tissus fixés des CSC par immunohistochimie (IHC)

L'utilisation de tissu fixé pour détection *in situ* des CSC est une technique plus facilement diffusable et plus pratique qu'un marquage nécessitant du tissu frais.
Les blocs fixés seront collectés afin de pouvoir effectuer des marquages *in situ* en utilisant des marqueurs IHC paraffine de CSC.

- L'anticorps ALDH1 est dirigé contre une des isoformes cytosoliques de l'aldéhyde déshydrogénase largement responsable de l'activité enzymatique détectée par le test ALDEFLUOR[82]. De plus, sa valeur pronostique péjorative a été démontrée sur ~500 carcinomes mammaires par le travail collaboratif avec l'équipe de M. Wicha. Elle présente l'avantage de pouvoir être effectuée à titre rétrospectif.

De façon optimale, les marquages seront comparés en pré, per et post-traitement sur la tumeur.
- la quantité de cellules ALDH1+ avec celle de CSC purifiées en cytométrie sur tumeur fraîche
- évaluer l’évolution de la proportion de ces cellules sous traitement
- corréler avec le phénotype CD44/CD24, associé lui aussi aux cellules souches cancéreuse dans le cancer du sein.

- **Les étapes méthodologiques** sont simples et le marquage utilise le kit Peroxydase Histostain plus (Zymed, South San Francisco, CA, USA) :
- déparaffinage des lames et réhydratation dans des bains graduels d’alcool,
- démasquage au tampon citrate pH6,
- incubation avec l’anti-ALDH1 (BD Biosciences) dilué au 1/100eme durant 1 heure,
- révélation colorimétrique à l’AEC (Zymed), contre–coloration (hématoxyline) et montage aqueux.

ANNEXE 4 : ETUDES ANCILLAIRES

1. CORRELATIONS ALDH1 ET CD44/CD24 EN IHC

Le marquage CD44 et CD24 va se faire par double marquage sur des sections de tumeurs adjacentes. Le traitement des lames est identique à celui effectué pour ALDH1. Le démasquage antigénique est fait à pH9 avec du tampon acide Tris-ethylenediamine tetraacetic. Le double marquage se fait dans un Automate (Dako) en utilisant le système Envision G|2 Doublestain System Rabbit/Mouse (DAB+/Permanent Red) (DAKO) selon les recommandations du fabriquant.
Les anticorps utilisés sont de Neomarkers (Fremont, CA, USA). La détection de l'anti-CD44 se fait en permanent Red et du CD24 en diaminobenzidine (DAB). On évalue la proportion de cellules CD44+/CD24- qui est associée au phénotype cellule souche par rapport à toutes les autres (CD44+/CD24+, CD44-/24+, CD44-/CD24-).
Cette proportion sera corrélée avec les autres méthodes de détection (IHC ALDH1 et test ALDEFLUOR).

2. MONITORING DES CELLULES TUMORALES CIRCULANTES PAR SYSTEME CELL SEARCH, VERIDEX

Objectifs

- Décrire les variations des niveaux de CTC (Circulating Tumour Cells) ~~et des CEC~~ (Circulating Endothelial Cells) entre les prélèvements baseline, et le J22 (avant la deuxième injection du traitement) ~~et avant la chirurgie~~
- Evaluer la valeur prédictive des variations des CTC ~~et CEC~~ sur la réponse histologique, la survie sans maladie, la survie sans rechute et la survie globale
- Evaluer la corrélation entre taux de CTC ~~et CEC~~ et le taux initial et sous traitement de CSC

Prélèvements : ~~2 tubes~~ 1 tube de 10 ml de sang total pour traitement immédiat (recherche de CTC ~~et CEC~~ système Veridex®, CellSearch), à envoyer en frais à :
Pr Jean-Yves PIERGA/
Dr Claire MATHIOT
Laboratoire d'Hématologie
Institut Curie
26 Rue d'Ulm
75005 PARIS

Critères de jugement

- Niveaux de CTC ~~et CEC~~ avant traitement, et à J22 ~~et avant la chirurgie~~
- Survie sans maladie, survie sans rechute, survie globale à 3 et 5 ans
- Réponse complète histologique
- Taux initial et à 12 semaines de CSC

Rationnel

La présence de cellules tumorales circulantes (CTC) dans le sang de patientes présentant un cancer du sein métastatique, soit par des techniques de biologie moléculaire telles que la RT-PCR[2], soit par des techniques d'immunomarquage[3], est associée à un très mauvais pronostic[1]. Les techniques de PCR sont des techniques très sensibles mais souvent peu spécifiques[5, 6]. Les marqueurs utilisés pour une telle détection sont des glycoprotéines membranaires ou des constituants du cytosquelette comme les cytokératines[4]. Une étape d'enrichissement, via une sélection positive ou négative en utilisant des sphères immuno-magnétiques, est recommandée dans le but de d'éliminer le bruit de fond et d'augmenter le nombre de cellules tumorales détectées dans l'échantillon[7]. Le monitoring de ces cellules tumorales circulantes pourrait permettre de prédire précocement le bénéfice d'un traitement anti-angiogénique. De plus, de nombreuses études ont suggéré que le niveau de cellules endothéliales circulantes (CEC) était élevé dans le sang de patients atteints de cancer et que

l'évolution du nombre de ces cellules pouvait être associée à la réponse aux traitements anti-angiogéniques (9-13).
La technique Cellsearch (Veridex®) sera utilisée sur sang frais pour mesurer ~~à la fois~~ les CTC ~~et les CEC~~ et corréler le niveau mesuré en base-line ainsi que son évolution après le premier cycle, à la réponse thérapeutique et à l'évolution clinique.

Références

1. Seronie-Vivien S, Mery E, Delord JP, Fillola G, Tkaczuk J, Voigt JJ, et al.Carcinocythemia as the single extension of breast cancer: report of a case and review of the literature. Ann Oncol 2001;12(7):1019-22.
2. Ring A, Smith IE, Dowsett M. Circulating tumour cells in breast cancer. Lancet Oncol 2004;5(2):79-88.
3. Braun S, Naume B. Circulating and disseminated tumor cells. J Clin Oncol 2005;23(8):1623-6.
4. de Crémoux P, Extra JM, Denis MG, Pierga JY, Bourstyn E, Nos C, et al. Detection of MUC1-expressing mammary carcinoma cells in the peripheral blood of breast cancer patients by real-time polymerase chain reaction. Clin Cancer Res 2000;6(8):3117-22.
5. Jain RK, Duda DG, Clark JW, Loeffler JS. Lessons from phase III clinical trials on anti-VEGF therapy for cancer. Nat Clin Pract Oncol 2006;3(1):24-40.
6. Mancuso P, Rabascio C, Bertolini F. Strategies to investigate circulating endothelial cells in cancer. Pathophysiol Haemost Thromb 2003;33(5-6):503-6.
7. Mancuso P, Burlini A, Pruneri G, Goldhirsch A, Martinelli G, Bertolini F. Resting and activated endothelial cells are increased in the peripheral blood of cancer patients. Blood 2001;97(11):3658-61.Protocol ML 21531 - Version n° 2 of 26 April 2008 98 / 113.
8. Mancuso P, Colleoni M, Calleri A, Orlando L, Maisonneuve P, Pruneri G, et al. Circulating endothelial-cell kinetics and viability predict survival in breast cancer patients receiving metronomic chemotherapy. Blood 2006;108(2):452-9.
9. Willett CG, Boucher Y, di Tomaso E, Duda DG, Munn LL, Tong RT, et al. Direct evidence that the VEGF-specific antibody bevacizumab has antivascular effects in human rectal cancer. Nat Med 2004;10(2):145-7.
10. Willett CG, Boucher Y, Duda DG, di Tomaso E, Munn LL, Tong RT, et al. Surrogate markers for antiangiogenic therapy and dose-limiting toxicities for bevacizumab with radiation and chemotherapy: continued experience of a phase I trial in rectal cancer patients. J Clin Oncol 2005;23(31):8136-9.
11. Beaudry P, Force J, Naumov GN, Wang A, Baker CH, Ryan A, et al. Differential effects of vascular endothelial growth factor receptor-2 inhibitor ZD6474 on circulating endothelial progenitors and mature circulating endothelial cells: implications for use as a surrogate marker of antiangiogenic activity. Clin Cancer Res 2005;11(9):3514-22.
12. Cristofanilli M, Budd GT, Ellis MJ, Stopeck A, Matera J, Miller MC, et al. Circulating tumor cells, disease progression, and survival in metastatic breast cancer. N Engl J Med 2004;351(8):781-91.
13. Cristofanilli M, Hayes DF, Budd GT, Ellis MJ, Stopeck A, Reuben JM, et al. Circulating tumor cells: a novel prognostic factor for newly diagnosed metastatic breast cancer. J Clin Oncol 2005;23(7):1420-30.

3. TRANSCRIPTOMIQUE

Objectifs

Une étude transcriptionnelle sera réalisée sur puces à ADN pangénomique à partir des échantillons tumoraux obtenus avant traitement.
Sera évaluée la valeur prédictive des profils d'expression ARNm pré-thérapeutique sur la réponse histologique, la survie sans maladie, la survie sans rechute et la survie globale, ainsi que leur corrélation avec le taux de CSC et leur évolution sous traitement.

Critères de jugement

- Profils d'expression ARNm sur puces pan génomiques avant traitement (macrobiopsie initiale)
- Réponse histologique, survie sans maladie, survie sans rechute et survie globale à 3 et 5 ans
- Taux initial et à 12 semaines de CSC

Rationnel

Jusqu'à récemment, les analyses moléculaires ont souvent été focalisées sur un ou quelques gènes à la fois, apportant une information spécifique mais souvent biologiquement limitée. Les techniques d'analyses moléculaires à haut débit et à grande échelle devraient permettre de mieux appréhender la complexité moléculaire des cancers à l'origine de leur grande hétérogénéité clinique.
Un grand nombre d'études récentes utilisant l'analyse des profils d'expression génique par la technique des puces à ADN ont démontré les résultats prometteurs de cette approche dans le cancer du sein en mettant en évidence de nouveaux sous-types moléculaires de pronostique distinct, non identifiables par les paramètres conventionnels ainsi que de nouvelles signatures moléculaires dont l'impact pronostique et prédictif pourrait être supérieur aux paramètres histocliniques conventionnels.
Ces données justifient l'évaluation de ces technologies pour déterminer un profil d'expression génique associé à la sensibilité au bevacizumab et/ou au taux initial de CSC.

Références

1. Perou CM, Jeffrey SS, van de Rijn M, et al: Distinctive gene expression patterns in human mammary epithelial cells and breast cancers. Proc Natl Acad Sci U S A 96:9212-7, 1999
2. Golub TR, Slonim DK, Tamayo P, et al: Molecular classification of cancer: class discovery and class prediction by gene expression monitoring. Science 286:531-7, 1999
3. Perou CM, Sorlie T, Eisen MB, et al: Molecular portraits of human breast tumours. Nature 406:747-52, 2000
4. Sorlie T, Perou CM, Tibshirani R, et al: Gene expression patterns of breast carcinomas distinguish tumor subclasses with clinical implications. Proc Natl Acad Sci U S A 98:10869-74, 2001
5. Bertucci F, Nasser V, Granjeaud S, et al: Gene expression profiles of poor-prognosis primary breast cancer correlate with survival. Hum Mol Genet 11:863-72, 2002
6. van 't Veer LJ, Dai H, van de Vijver MJ, et al: Gene expression profiling predicts clinical outcome of breast cancer. Nature 415:530-6, 2002
7. Sotiriou C, Neo SY, McShane LM, et al: Breast cancer classification and prognosis based on gene expression profiles from a population-based study. Proc Natl Acad Sci U S A 100:10393-8, 2003
8. Sorlie T, Tibshirani R, Parker J, et al: Repeated observation of breast tumor subtypes in independent gene expression data sets. Proc Natl Acad Sci U S A 100:8418-23, 2003
9. Bertucci F, Viens P, Hingamp P, et al: Breast cancer revisited using DNA array-based gene expression profiling. Int J Cancer 103:565-71, 2003
10. Bertucci F, Viens P, Tagett R, et al: DNA arrays in clinical oncology: promises and challenges. Lab Invest 83:305-16, 2003
11. Bertucci F, Borie N, Ginestier C, et al: Identification and validation of an ERBB2 gene expression signature in breast cancers. Oncogene 23:2564-75, 2004
12. Rouzier R, Perou CM, Symmans WF, et al: Breast Cancer Molecular Subtypes Respond Differently to Preoperative Chemotherapy. Clin Cancer Res 11:5678-5685, 2005
13. Bertucci F, Finetti P, Cervera N, et al: Gene expression profiling shows medullary breast cancer is a subgroup of basal breast cancers. Cancer Res 66:4636-44, 2006

4. PROTEOMIQUE

Objectifs

Une étude protéomique plasmatique et tissulaire en spectrométrie de masse (SELDI-TOF MS précédée d'une «équalisation» et/ou d'une immunodéplétion puis identification par Orbitrap) sera réalisée à partir des échantillons biologiques obtenus avant et pendant le traitement.

Sera évaluée la valeur prédictive des profils d'expression protéique sur la réponse histologique, la survie sans maladie, la survie sans rechute et la survie globale, ainsi que leur corrélation avec le taux de CSC et leur évolution sous traitement.

Critères de jugement

- Profils d'expression protéiques avant et sous traitement (macrobiopsie initiale, prélèvements sanguins répétés)
- Réponse histologique, survie sans maladie, survie sans rechute et survie globale à 3 et 5 ans
- Taux initial et à 12 semaines de CSC

Rationnel

Dans le contexte de développement des approches en «omics», les approches protéomiques sont parmi les outils les plus récemment développés pour identifier des biomarqueurs. De façon similaire aux études visant à décrire les altérations génomiques et/ou transcriptionnelles associées aux cancers et à leurs aspects évolutifs ou à leur réponse thérapeutique spécifique, les différents outils d'analyses du protéome permettent l'étude globale et comparative des protéines au sein d'échantillons caractérisés sur le plan biologique et clinique. Les technologies basées sur la spectrométrie de masse fonctionnent sans contrainte initiale sur une connaissance «a priori» et peuvent donc examiner et quantifier un grand nombre de paramètres protéiques initialement inconnus parmi lesquels un ou plusieurs candidats à identifier secondairement peuvent corréler avec un phénotype tumoral d'intérêt. Les échantillons biologiques pouvant faire l'objet de ce type d'analyse incluent les fluides biologiques, et notamment sérum ou plasma, d'un intérêt tout particulier pour la recherche de biomarqueurs en condition clinique ainsi que les tissus tumoraux congelés voire fixés et inclus en paraffine. Il apparaît donc possible et prometteur d'appliquer ces technologies à des échantillons cliniques ou pré-cliniques correctement annotés afin d'identifier des biomarqueurs protéiques théragnostiques innovants en oncologie. Les projets de protéomique clinique ont été boostés ces dernières années par l'utilisation de la technologie de spectrométrie de masse SELDI-TOF (surface enhanced laser desorption ionization-time of flight). Cette approche permet l'analyse d'échantillons biologiques hautement complexes avec un relatif haut débit et un minimum d'étapes pré-analytiques ; elle combine fractionnement chromatographique du protéome utilisant des puces à protéines (proteinchips arrays) et spectrométrie de masse TOF et elle peut être appliquée aux fluides biologiques tels que le sérum ou plasma comme aux tissus.
Au niveau sérique, nous avons récemment décrit pour la première fois que le profil protéique sérique pouvait porter une information pronostique chez des patientes atteintes d'un cancer du sein localisé avec des facteurs de mauvais pronostic. En utilisant la technologie SELDI-TOF, nous avons rétrospectivement analysé le sérum post-opératoire précoce de 81 patientes présentant un cancer du sein à haut risque avec une indication de chimiothérapie adjuvante. Les sérums collectés après chirurgie et avant initiation de la chimiothérapie adjuvante ont été fractionnés en combinant billes échangeuses d'anions et rétention sur des ProteinChips de différentes spécificités chromatographiques, puis analysés en spectrométrie de masse. Les protéines différentiellement exprimées en fonction de l'évolution clinique (rechute métastatique ou non) ont été combinées au sein d'un modèle de régression logistique permettant de générer un index protéique de 40 protéines capable de prédire correctement l'évolution métastatique dans 83% des cas et distinguant 2 groupes de patients avec des survies sans rechute métastatique et des survies globales radicalement différentes. Cet index apparaissait en analyse multivariée comme le paramètre pronostique indépendant

le plus significativement associé à la probabilité de rechute. L'identification de certaines des protéines constitutives de cet index a été réalisée et celles-ci correspondent essentiellement à des protéines de la réponse de l'hôte, potentiellement impliquées dans des processus biologiques pouvant influer sur le processus métastatique tels que la réponse immune ou le processus de néoangiogénèse.
Par ailleurs, des approches préliminaires ont concerné lignées cellulaires et tissus tumoraux provenant de cancers du sein dont les caractéristiques moléculaires et notamment l'appartenance aux différents sous types moléculaires avaient été précisées. Ainsi, il a été possible d'interroger en spectrométrie de masse le protéome de ces différents matériels et d'identifier plusieurs biomarqueurs associés au phénotype basal.
Ces données justifient l'analyse protéomique des échantillons biologiques (plasma et tumeur) collectés dans le cadre de cet essai dans le but d'identifier des paramètres prédictifs de la réponse thérapeutique et/ou des biomarqueurs associés au CSC.

Références

1. Sawyers, C. L. The cancer biomarker problem. Nature 452, 548-52 (2008).
2. National Institutes of Health. (September 8. Meeting summary report, Biomarkers Knowledge System. (2000).
3. Baselga, J. et al. Mechanism of action of trastuzumab and scientific update. Semin Oncol 28, 4-11 (2001).
4. Lievre, A. et al. KRAS mutation status is predictive of response to cetuximab therapy in colorectal cancer. Cancer Res 66, 3992-5 (2006).
5. Taguchi, F. et al. Mass spectrometry to classify non-small-cell lung cancer patients for clinical outcome after treatment with epidermal growth factor receptor tyrosine kinase inhibitors: a multicohort cross-institutional study. J Natl Cancer Inst 99, 838-46 (2007).
6. Goncalves, A. et al. Postoperative serum proteomic profiles may predict metastatic relapse in high-risk primary breast cancer patients receiving adjuvant chemotherapy. Oncogene 25, 981-9 (2006).
7. Goncalves, A. et al. Serum proteomic prediction of progression-free survival in HER2-negative metastatic breast cancer patients receiving docetaxel as first-line treatment. J Clin Oncol (Meeting Abstracts) 25, 1010- (2007).
8. Goncalves, A. et al. Protein profiling of human breast tumor cells identifies novel biomarkers associated with molecular subtypes. Mol Cell Proteomics, M700487-MCP200 (2008).
9. Ginestier, C. et al. ALDH1 Is a Marker of Normal and Malignant Human Mammary Stem Cells and a Predictor of Poor Clinical Outcome. Cell Stem Cell 1, 555-567 (2007).
10. Bertucci, F. et al. Proteomics of breast cancer: principles and potential clinical applications. Mol Cell Proteomics 5, 1772-86 (2006).
11. Bertucci, F. et al. Clinical proteomics and breast cancer: strategies for diagnostic and therapeutic biomarker discovery. Future Oncol 4, 271-87 (2008).

5. POLYMORPHISME VEGF

Objectifs

Décrire la distribution des polymorphismes du VEGF (1 tube de 5 ml de sang) dans la population de l'étude et étudier les corrélations entre ces polymorphismes et la réponse complète histologique, la survie sans maladie, la survie sans rechute, la survie globale et la survenue d'effets secondaires.

Critères de jugement

- Recherche des polymorphismes du VEGF -2578C/A, -1154G/A, +936C/T, -634G/C
- Corrélation avec la réponse histologique, la survie sans maladie, la survie sans rechute et la survie globale à 3 et 5 ans.

Rationnel

Plusieurs polymorphismes du gène VEGF ont été écrits et quatre sont relativement fréquents pouvant influencer l'effet du VEGF sur le développement des tumeurs solides. Deux de ces polymorphismes sont situés dans la région promotrice en 2578C/A et -1154G/A, un est localisé dans la région 3' non transcrite (3'UTR) en +936C/T et un autre est en 5'UTR en -634G/C. Les allèles -2578C, -1154G et -634C (30%) ont été associés avec une expression plus élevée de VEGF tandis que l'allèle +936T (16%) corrèle avec une plus faible expression du VEGF et peut être un plus faible risque de cancer du sein. Certains génotypes et haplotypes du VEGF pourraient avoir un effet sur la croissance tumorale, le processus métastatique et la survie. Ainsi, les allèles -2578C et -634C semblent corrélés à un plus haut grade histologique. Enfin, très récemment, certains polymorphismes du VEGF ont pu être associés à un bénéfice de survie pour l'association taxol bevacizumab dans les cancers du sein métastatiques.

Références

1. Schneider BP, Radovich M, Sledge GW, et al: Association of polymorphisms of angiogenesis genes with breast cancer. Breast Cancer Res Treat [epub ahead of print on September 20, 2007]
2. Krippl P, Langsenlehner U, Renner W, et al: A common 936 C/T gene polymorphism of vascular endothelial growth factor is associated with decreased breast cancer risk. Int J Cancer 106:468-471, 2003[CrossRef][Medline]
3. Jacobs EJ, Feigelson HS, Bain EB, et al: Polymorphisms in the vascular endothelial growth factor gene and breast cancer in the Cancer Prevention Study II cohort. Breast Cancer Res 8:R22, 2006[CrossRef][Medline]
4. Jin Q, Hemminki K, Enquist K, et al: Vascular endothelial growth factor polymorphisms in relation to breast cancer development and prognosis. Clin Cancer Res 11:3647-3653, 2005[Abstract/Free Full Text]
5. Schneider BP, Skaar TC, Sledge GW, et al: Analysis of angiogenesis genes from paraffin-embedded breast tumor and lymph nodes. Breast Cancer Res Treat 96:209-215, 2006[CrossRef][Medline]
5. Schneider BP, Wang M, Radovich M, et al: Association of Vascular Endothelial Growth Factor and Vascular Endothelial Growth Factor Receptor-2 Genetic Polymorphisms With Outcome in a Trial of Paclitaxel Compared With Paclitaxel Plus Bevacizumab in Advanced Breast Cancer: ECOG 2100. J Clin Oncol 26:4672-4678, 2008

6. ECHOGRAPHIE MAMMAIRE AVEC INJECTION DE PRODUIT DE CONTRASTE

Objectifs

Décrire les modifications de la néoangiogenèse tumorale en échographie avec injection de produit de contraste sous traitement et suggérer les modifications précoces permettant d'identifier les répondeurs au bevacizumab.

Critères de jugement

Modifications de la cinétique et de l'intensité de la prise de contraste en échographie avec injection de produit de contraste.
Corrélation à la réponse histologique, la survie sans maladie, la survie sans rechute, la survie globale et les variations du taux de CSC, de CTC et de CEC.

Rationnel

La réponse thérapeutique aux traitements néo-adjuvants dans les cancers du sein est évaluée en mammographie et en échographie mammaire. Il s'agit cependant d'une réponse exclusivement morphologique basée sur les modifications de taille de la tumeur qui ne reflète pas forcément l'activité biologique du traitement entrepris, notamment en matière de nouvelles thérapeutiques anti-angiogéniques. Avec ces composés, il a été montré que les modifications de la vascularisation peuvent précéder les modifications morphologiques. L'évaluation de la réponse thérapeutique à ces composés devrait donc prendre en compte ces éléments. Parmi les méthodes d'imagerie fonctionnelle, l'échographie à produit de contraste avec injection de microbulles est une technique non invasive, simple et reproductible qui pourrait permettre d'identifier précocement les répondeurs à une thérapeutique anti-angiogénique.

Références

1. Therasse P, Arbruck SG, Eisenhaur EA, et al. New guidelines to evaluate the response to treatment in solid tumors. European Organization for Research and Treatment of Cancer Institute of the United States, National Cancer Institute of Canada. J Natl Cancer Inst 2000; 92:205-16.
2. Schwartz L. Conventional and novel techniques for therapeutic response assessment. Radiological Society North America (RSNA). 90th scientific assembly and annual meeting, Chicago. Proc Radiology 2004:115.
3. Benjamin RS, Choi H, Macapinlac HA, Burgess MA, Patel SR, Chen LL, et al. We should desist using Recist, at least in gastrointestinal stromal tumors. J Clin Oncol 2007; 25:1760-4.
4. De giorgi U, Aliberti C, Benea G, Conti M, Marangolo M. Effect of angiosonography to monitor response during imatinib treatment in patients with metastatic gastrointestinal stromal tumors. Clin Cancer Res 2005;11(17):6171-76.
5. Krix M, Kiessling F, Vosseler S, et al. Sensitive noninvasive monitoring of tumor perfusion during antiangiogenic therapy by intermittent bolus-contrast power Doppler sonography. Cancer Res 2003;63:8264-70.
6. Aramand JP, Loriot Y, Ropert S, Catteay A, Soria JC, Lassau N. Perfusion assessment of tumors by Doppler ultrasonography, a tool for early evaluation of targeted antiangiogenic compounds. Ann Oncol 2006;17(suppl3):iii21.
7. Lassau N, Roche A. Imagerie et angiogenèse: la DCE-US (dynamic contrast enhancedultrasonography). Bull Cancer 2007; 94 :S247-53.

ANNEXE 5 : GESTION DES TOXICITÉS INDUITES PAR LE BEVACIZUMAB

Adverse Event	Grade CTCAE v3.0	Action to be Taken
**Infusion related or Allergic reaction/ Acute hypersensitivity reaction (e.g., fever, rash, urticaria, bronchospasm)**	1, 2, or 3	- *If infusion-related or allergic reactions occur, pre-meds should be given with the next dose*, but the infusion time may not be ↓ for the subsequent infusion. If the next dose is well-tolerated with pre-meds, the subsequent infusion time may be ↓ by 30 ± 10 min. as long as pre-meds continue to be used. If infusion-related AEs occur with the 60-min. infusion, all subsequent doses should be given over 90 ± 15 min. (with pre-meds). If infusion-related AEs occur with the 30-min. infusion, all subsequent doses should be given over 60 ± 10 min. (with pre-meds).<br>- *For patients with grade 3 reactions*, the bevacizumab infusion should be stopped and not re-started on that day. At the physician's discretion, bevacizumab may be permanently discontinued or re-instituted with pre-medications and at a rate of 90 ± 15 minutes. If the reaction occurred at the 90-minute rate, initially challenge at a slower infusion rate and gradually increase to 90 min. When bevacizumab is re-instituted, the patient should be monitored per physician's usual practice for duration comparable to duration of reaction.
	4	Permanently discontinue bevacizumab.
**Hypertension**	2 or 3	Bevacizumab may be continued in conjunction with standard anti-hypertensive therapy at the physician's discretion. Bevacizumab may continue with systolic BP of 140-150 mmHg ***but anti-hypertensive therapy should be initiated or intensified***. Bevacizumab should be held for systolic BP > 150 mmHg or diastolic BP > 90 mmHg present on the day that the bevacizumab dose is to be given. Medication to control hypertension should be initiated if bevacizumab is held. If BP is not controlled with medication within 6 weeks, permanently discontinue bevacizumab.
	4	Permanently discontinue bevacizumab.
**Hemorrhage**	3 or 4	Permanently discontinue bevacizumab. ***(Note: Bevacizumab must also be permanently discontinued for grade 2 pulmonary or CNS hemorrhage.)***
**Thrombosis/embolism-venous (including vascular access device)**	2 or 3	Hold bevacizumab until clinical resolution.<br>– Hold bevacizumab during 2 weeks AND<br>– Start full dose anticoagulation therapy.<br>After 2 weeks, bevacizumab may be resumed during anticoagulation therapy IF:<br>- Stable dose of anticoagulation therapy AND coagulation testing shows results within the therapeutic range.<br>- No grade 3 or 4 hemorrage event occurred while on therapy
	4	Permanently discontinue bevacizumab.
**Visceral or peripheral arterial ischemia**	2, 3, or 4	Permanently discontinue bevacizumab.
**CNS ischemia**	3 or 4	Permanently discontinue bevacizumab.
**Signs of Reversible Posterior Leuco encephalopathy Syndrome (RPLS)**	≥ 1	For clinical features suggestive of RPLS, hold bevacizumab and obtain MRI. If RPLS is diagnosed ***or*** if ANY symptoms were grade 4, permanently D/C bevacizumab. If RPLS is not diagnosed, bevacizumab may be resumed when presenting symptoms ≤ grade 1 ***and*** blood pressure < 150/90 mmHg.
**GI perforation including GI leak and GI fistula**	≥ 1	Permanently discontinue bevacizumab.
**Non-GI perforation including non-GI fistula**	≥ 1	Permanently discontinue bevacizumab.
**Intra-abdominal abscess[c]**	3	Hold bevacizumab until resolved.
	4	Permanently discontinue bevacizumab.
**Wound healing complications**	1	Hold bevacizumab for at least 3 weeks. If, in the physician's opinion, substantial healing has taken place within 1-3 months, bevacizumab may be resumed. If wound dehiscence recurs, permanently discontinue bevacizumab.
	2, 3, or 4	Permanently discontinue bevacizumab.
**Proteinuria[e]**	Cf Annexe 5	
**Other clinically significant adverse events[f]**	3	Hold until AE has resolved to ≤ grade 1.
	4	Permanently discontinue bevacizumab.

c Refer to grading criteria listed for the appropriate AE in the Infection Section of the CTCAE v 3.0.
d Refer to Dermatology/Skin Section of the CTCAE v 3.0.
e CTCAE v3.0 criteria for grading proteinuria in g/24 hours will be estimated by use of dipstick testing; ***24-hour urine collection is required for ≥ grade 2 (dipstick 2+ to 3***+). For more details, see Annexe 5
f Determination of "clinically significant" is at the physician's discretion and applies to those ***AEs that are not clearly associated with chemotherapy or with trastuzumab and could be related to bevacizumab.***

GESTION DES TOXICITÉS CARDIAQUES INDUITES PAR LE BEVACIZUMAB

Management of Congestive Heart Failure (CHF) during Bevacizumab and Trastuzumab treatment – LVEF assessment.

Note: Trastuzumab and bevacizumab **must** be discontinued in patients who have a **symptomatic** decrease in LVEF.

Instructions for symptomatic decrease in LVEF

- *Grade 3 CHF:* Patients should be monitored for signs and symptoms of CHF i.e., dyspnea, tachycardia, cough, neck vein distention, cardiomegaly, hepatomegaly, paroxysmal nocturnal dyspnea, orthopnea, peripheral edema, etc. **If the patient develops any of these signs and symptoms, trastuzumab and bevacizumab must be held.**

The investigator must confirm the diagnosis of CHF either with an echocardiogram or MUGA scan. **Once the diagnosis of CHF is confirmed, trastuzumab and bevacizumab must be permanently discontinued** and all reports must be submitted with the cardiac event report form and the LVEF assessment form within 21 days of the LVEF assessment.

- *Grade 4 CHF (severe refractory CHF or requiring intubation)*: **Discontinue trastuzumab and bevacizumab.**

Instructions for asymptomatic decrease in LVEF

- In case of asymptomatic decrease in LVEF > or equal to 50% following LVEF assessment during study therapy, targeted therapies* has to be continued.
- In case of asymptomatic decrease in LVEF < 50% following LVEF assessment during study therapy or absolute decrease of 15% compared to the value of the base, bevacizumab must be discontinued.

** targeted therapies: bevacizumab and trastuzumab*

Note that bevacizumab should be temporarily interrupted in the event of febrile Grade 4 neutropenia and/or Grade 4 thrombocytopenia, since these conditions are predisposing factors for increased bleeding tendency.

GESTION DES PROTÉINURIES INDUITES PAR LE BEVACIZUMAB

1) First occurrence of proteinuria during treatment with bevacizumab (algorithm 1 below)

• **< 2+** proteinuria (dipstick): administer bevacizumab as planned

• **≥ 2+** proteinuria (dipstick): administer bevacizumab as planned and collect 24-hour urine for determination of total protein within 3 days before the next scheduled bevacizumab administration:

- **If 24-hour proteinuria ≤ 2 g**: administer next bevacizumab dose as scheduled

- **If 24-hour proteinuria > 2 g**: omit next scheduled bevacizumab dose and do 24-hour urine collection for determination of total protein within 3 days before the subsequently scheduled cycle. Delay bevacizumab treatment until proteinuria has decreased to ≤2 g. Do 24-hour urine before each scheduled dose until proteinuria has improved to ≤1 g/24 hours, but omit bevacizumab only if > 2 g/24 hours

• Nephrotic syndrome (Grade 4, NCI CTC-AE v3.0): Permanently discontinue bevacizumab treatment.

Algorithm 1 for proteinuria (dipstick) 2+

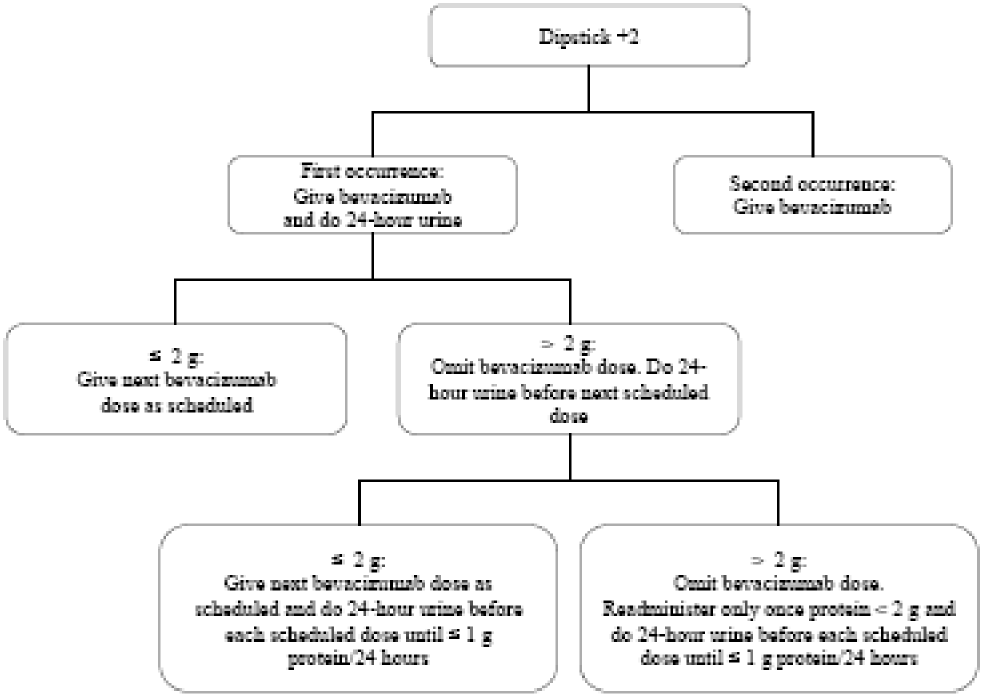

2) Second and subsequent occurrence of proteinuria during treatment with bevacizumab

(algorithm 2 below)

• **< 3+** proteinuria (dipstick): administer bevacizumab as planned

• **≥ 3+** proteinuria (dipstick): administer bevacizumab as planned and collect 24-hour urine for determination of total protein within 3 days before the next scheduled bevacizumab administration:

- **If 24-hour proteinuria ≤ 2 g**: administer next bevacizumab dose as scheduled

- **If 24-hour proteinuria > 2 g**: omit next scheduled bevacizumab dose and do 24-hour urine collection for determination of total protein within 3 days before the subsequently scheduled cycle. Delay bevacizumab treatment until proteinuria has decreased to ≤2 g. Do 24-hour urine before each scheduled dose until proteinuria has improved to ≤1 g/24 hours, but omit bevacizumab only if > 2 g/24 hours

• Nephrotic syndrome (Grade 4, NCI CTC-AE v3.0): Permanently discontinue bevacizumab Treatment

<u>Algorithm for proteinuria (dipstick) 3+</u>

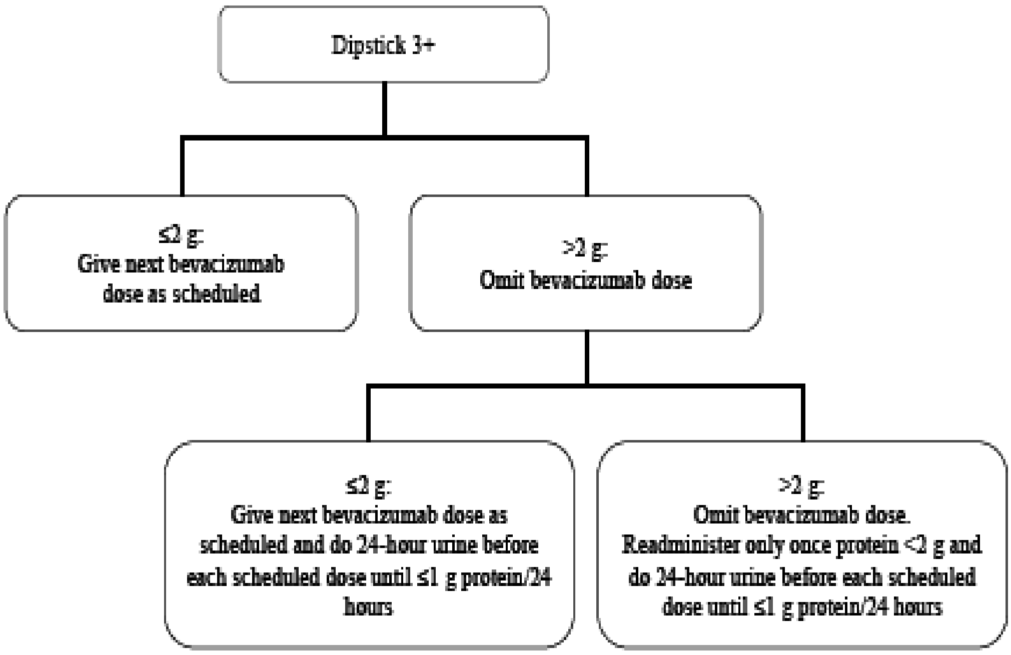

ANNEXE 6 : ECHELLE DE TOXICITE NCI CTC-AE v3.0

Se référer à l'échelle d'évaluation de la toxicité
" COMMON TERMINOLOGY CRITERIA FOR ADVERSE EVENTS " (CTCAE) version 3.0
que l'on peut télécharger sur le site du National Cancer Institute (NCI)

NATIONAL CANCER INSTITUTE

Cancer Therapy Evaluation Program

http://ctep.info.nih.gov/reporting/ctc.html

CTCAE v3.0 includes Adverse Events applicable to all oncology clinical trials regardless of chronicity or modality.

ANNEXE 7 : Classification de la NYHA

Clinical Evaluation of Functional Capacity of Patients
with Heart Disease in Relation to Ordinary Physical Activity

NYHA	Functional Class	Description	Objective Assessment
I	Mild	No limitation of physical activity. Ordinary physical activity does not cause undue fatigue, palpitation or dyspnoea.	No objective evidence of cardiovascular disease.
II	Mild	Slight limitation of physical activity. Comfortable at rest, but ordinary physical activity results in fatigue, palpitation or dyspnoea	Objective evidence of minimal cardiovascular disease
III	Moderate	Marked limitation of physical activity. Comfortable at rest, but less than ordinary activity causes fatigue, palpitation or dyspnoea.	Objective evidence of moderately severe cardiovascular disease.
IV	Severe	Unable to carry out any physical activity without discomfort. Symptoms of cardiac insufficiency at rest. If any physical activity is undertaken, discomfort is increased.	Objective evidence of severe cardiovascular disease.

The Criteria Committee of the New York Heart Association. *Nomenclature and Criteria for Diagnosis of Diseases of the Heart and Great Vessels*. 9th ed. Boston, Mass: Little, Brown & Co; 1994:253-256

ANNEXE 8 : RÉSUMÉ DES CARACTÉRISTIQUES DES PRODUITS

Se référer aux dernières versions en vigueur sur le site des agences réglementaires Afssaps, EMEA ou sur le site du VIDAL :

http://agmed.sante.gouv.fr/

http://www.emea.eu.int/

http://www.vidalpro.net/
