# Supplementary material for: Stem Cells Inhibition by Bevacizumab in Combination with Neoadjuvant Chemotherapy for Breast Cancer
Source: J Clin Med. 2019 May 6;8(5):612. doi: 10.3390/jcm8050612 (PMC6572380; doi:10.3390/jcm8050612)
Supplement: Supplementary file 1 [file jcm-08-00612-s001.zip › AVASTEM_SupData-JClinMed.pdf]

SUPPLEMENTARY DATA

AVASTEM – Stem cells inhibition by Bevacizumab in combination with neoadjuvant chemotherapy for breast cancer: a prospective proof of concept randomized phase II trial.

R Sabatier[1,2]; E Charafe-Jauffret[2,3]; JY Pierga[4]; H Curé[5]; E Lambaudie[2,6]; D Genre[7], G Houvenaeghel[2,6]; P Viens[1,2]; C Ginestier[2]; F Bertucci[1,2]; P Sfumato[8]; JM Extra[1*]; A Gonçalves - MD, PhD[1,2*].

1. Department of Medical Oncology, Institut Paoli-Calmettes, Marseille, France
2. Aix Marseille Univ, CNRS U7258, INSERM U1068, Institut Paoli-Calmettes, CRCM, Marseille, France
3. Department of Biopathology, Institut Paoli-Calmettes, Marseille, France
4. Department of Medical Oncology, Institut Curie, Paris & St Cloud, Université Paris Descartes, France
5. Department of Medical Oncology, Institut Jean Godinot, Reims, France
6. Department of Surgical Oncology, Institut Paoli-Calmettes, Marseille, France
7. Department of Clinical Research and Innovation, Institut Paoli-Calmettes, 13009 Marseille, France
8. Department of Clinical Research and Innovation, Biostatistics unit, Institut Paoli-Calmettes, 13009 Marseille, France

*These authors contributed equally to this work

Supplementary tables: Cox regression multivariate analyses

Table S1: Overall survival for the whole population

		Hazard Ratio [95CI]	*p-value*
Treatment arm	Bevacizumab *vs*. Control	2.59 [0.30-22.6]	0.39
ALDH1 at inclusion	negative *vs*. positive	0.52 [0.10-2.78]	0.44
Pathological response	RD *vs*. pCR	1.18 [0.19-7.09]	0.86

Table S2: Disease-free survival for M0 patients

		Hazard Ratio [95CI]	*p-value*
Treatment arm	Bevacizumab *vs*. Control	0.89 [0.28-2.79]	0.84
ALDH1 at inclusion	negative *vs*. positive	0.96 [0.31-3.04]	0.95
Pathological response	RD *vs*. pCR	1.55 [0.45-5.33]	0.49

Table S3: Relapse-free survival for M0 patients

		Hazard Ratio [95CI]	*p-value*
Treatment arm	Bevacizumab *vs*. Control	1.09 [0.32-3.75]	0.89
ALDH1 at inclusion	negative *vs*. positive	1.18 [0.34-4.07]	0.79
Pathological response	RD *vs*. pCR	1.50 [0.42-5.33]	0.53

pCR: pathological complete response, RD: invasive residual disease in breast or lymph nodes
